# Phycochemistry and pharmacological significance of filamentous cyanobacterium *Spirulina* sp.

**DOI:** 10.1186/s40643-025-00861-0

**Published:** 2025-04-03

**Authors:** Sanjana Sabat, Shuvasree Bej, Surendra Swain, Ajit Kumar Bishoyi, Chita Ranjan Sahoo, Goutam Sabat, Rabindra Nath Padhy

**Affiliations:** 1 Central Research Laboratory, Institute of Medical Sciences, Siksha ‘O’ Anusandhan Deemed to Be University, Bhubaneswar, Odisha 751003 India; 2 Department of Botany and Biotechnology, Khallikote Unitary University, Berhampur, Odisha 760001 India

**Keywords:** *Spirulina* sp., Phycocompounds, Pharmaceutical, Health benefits

## Abstract

The cyanobacterium*, Spirulina* sp. is a photosynthetic blue-green alga with essential nutrients, vitamins nucleic acids, proteins, carbohydrates, fatty acids and pigments carotenes; and phycocyanins are the significant components having immunomodulatory, anti-inflammatory properties, which are used in food and cosmetics industries. *Spirulina* sp. can play an important role in human and animal nutrition for potential health benefits due to their phycochemical and pharmaceutical significance. This study highlights antibacterial, antifungal, antiviral, antioxidant, nephroprotective, cardioprotective, anticancer, neuroprotective, anti-aging, anti-inflammatory, and immunomodulatory properties. It highlights anti-anemic, antidiabetic, probiotic, anti-malarial, anti-obesity and weight loss, anti-genotoxicity, anti-thrombic, radioprotective, and detoxifying effects of *Spirulina* sp. Pharmaceutical studies indicate it may improve heart health and add to the treatment of diabetes, obesity and weight loss. It can play a major role in protecting the environment by recycling wastewater and providing food for humans and animals. *Spirulina* sp. can supply ingredients for aquaculture and agricultural feeds, pigments, antioxidants, and essential omega-3 oils, among other human health and wellness products. The amino acid of *Spirulina* is among the greatest qualititavely of any plant, even higher than that of soybean. Furthermore, cyanobacterium *Spirulina* sp. could be a future antimicrobial drug agent.

**Graphical Abstract:**

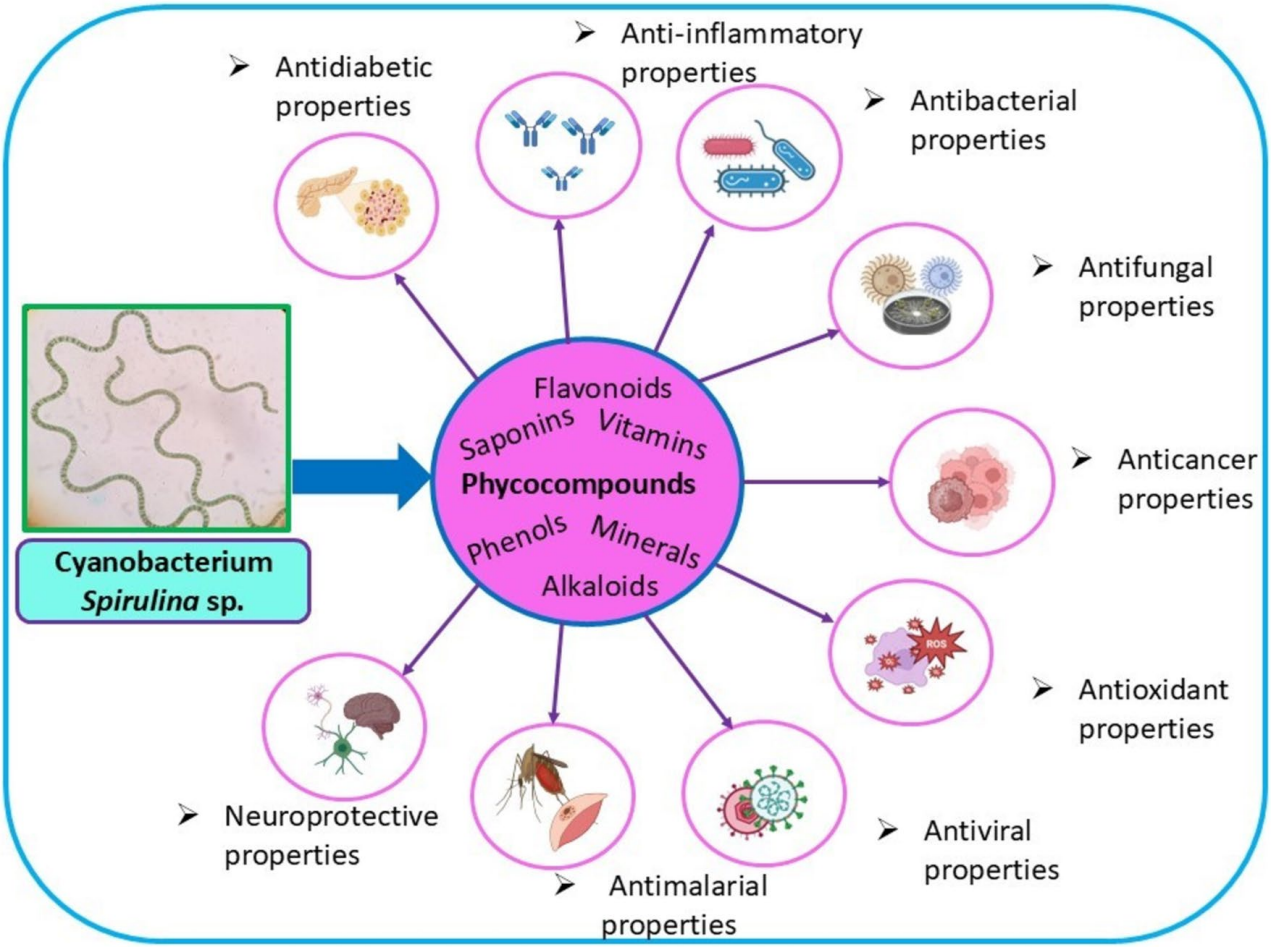

## Introduction

Cyanobacteria are Gram-negative prokaryotic organisms recognized for their potential to carry out oxygenic photosynthesis. These are seen in symbiotic relationships with lichen, bryophytes like hornworts, pteridophytes like *Azolla* sp*.*, cycads and angiosperms like *Gunnera* sp. (Adams et al. 2006; Nicoletti 2022; Chang et al. 2019; Bergman et al. 1992). Certain species of cyanobacteria can develop heterocyst to fix nitrogen, reducing atmospheric nitrogen into a combined form. The cells also have oxygenic photosynthesis in bacterial cell machinery. This cyanobacterium is present across a range of ecosystems, including inland water bodies, brackish water, marine water, geothermal springs, and basic saline environments (Vo et al. 2015). Less recently, phylogenetic analyses using 16S rRNA genes indicated that cyanobacteria represent a diverse, monophyletic phylum within the bacterial domain, underscoring their ecological and evolutionary significance (Engene et al. 2010). *Spirulina* sp., a non-nitrogen-fixing, non-heterocystous, filamentous and spiral-looking cyanobacterium (blue-green alga, or BGA), commonly known as green-ginseng for its abundance of proteins and amino acids as contents of curio; namely leucine, tryptophane, methionine, phenylalanine, lysine, thionine, isoleucine valine, etc. Its abundance in algal pigments, inherent antioxidants, and several natural pharmaceutics make it a demanding alga (Krishnan et al. 2024).

From phylogenetic view, *Arthrospira* sp. is positioned within the division Cyanobacteria, in the order Oscillatoriales, consisting of filamentous non-heterocystous cyanobacteria. Molecular studies, primarily through 16S rRNA sequencing, clarified its mis-belonging to the genus *Spirulina* sp.. With in the family Microcoleaceae, *Arthrospira* sp., have closer phylogenetic relationships toward other filamentous cyanobacteria that exhibited traits as trichomatous forms with gliding ability. Recent studies on the species of *Spirulina/Arthrospira*/*Limnospira* divide the strains into three clusters: planktonic Taxon I and II, and benthic Taxon III., respectively. However, the described and isolated species of helically coiled cyanobacteria have been under the genus *Arthrospira*, first reported by Stizenberger in 1852. Its classification has been controversial, in fact, Geitler in 1925 had invalidated the genus due to the absence of firm sheaths in the coiled species and included it within *Spirulina* in 1829 (Sili et al. 2012). However, this revision for decades was accepted until new research reinstated a clear distinction between species of *Spirulina* and *Arthrospira*. Thus, these evidences lead to reclassification of the most species back into *Arthrospira* sp. (Castenholz et al. 2001). Presently, three groups of cyanobacteria are distinguished: *Spirulina* sp. (12 species, with the type species *Spirulina subsalsa*), *Arthrospira* sp. (about 35 species, with the type species *Arthrospira jenneri*), and *Limnospira* sp. with 4 species, among them are *Limnospira fusiformis, Limnospira platensis, Limnospira maxima,* and *Limnospira indica*, being widely cultivated for biomass production. *Spirulina* and *Limnospira* sp. are not distinct genera. Instead, *Limnospira* sp. is a synonym of some species formerly classified under the genus *Spirulina* sp., mainly the strains that are commercially available. *Limnospira* sp. was created to reflect a better phylogenetic relationships of cyanobacteria, previously assigned to the genus *Arthrospira* (Hicks et al. 2021). Traditionally called *Spirulina maxima* or *Spirulina fusiformis*, belongs to the genus *Limnospira* sp., e.g., *Limnospira maxima, Limnospira platensis* and *Limnospira fusiformis* (Sinetova et al. 2024). Whole-genome sequencing further revealed that the genus *Limnospira* is essentially monospecific, Therefore, *L. platensis* PCC 7345 is the established reference strain, and other strains such as *L. maxima, L. fusiformis, L. indica* are now termed substrains of *L. platensis*. Initially, this organism was called *Spirulina oscillarioide*, and over the years, most commercially used species are now placed under the name *Limnospira* spp. due to molecular and morphological differences. This revision brings commercially important *Limnospira* species to differ from the type strain *A. jenneri* as well as according to recent phylogenetic studies. Yet, in commercial use, the term ‘spirulina’ is still used in referring to dried or living biomass of *Arthrospira/Spirulina /Limnospira*. These findings consolidate *L. platensis* as the primary source for scientific research and business purposes. However commercially grown material remains known as Spirulina, to be written in lowercase, without italics.

*Spirulina* have evolved in diverse morphological and biochemical environments, including freshwater, marine, and in rigorous conditions such as thermal springs and barren areas; furthermore, these BGA display diverse morphologies, with solitary cells forming aggregates or as benthic substrates to filamentous cyanobacteria that may appear as single trichomes or as bundles with varying thicknesses (Casamatta et al. 2016). Some filamentous forms are enclosed in a protective sheath, enhancing survival in unfavorable conditions. Heterotrophic flexibility and, occasionally, mixotrophic ability are crucial metabolic features of *Spirulina platensis* to sustain in unfavourable environmental conditions. Generally, under oxygenic photosynthesis *Spiruina* sp. expands its energy reserves using light, but in the absence or scarcity of it, heterotrophic metabolism is performed in which organic compounds such as glucose, sucrose or any organic acids are utilized. *Spirulina* sp. is also capable of anoxygenic photosynthesis or sulfur reduction, in which it replaces oxygen in metabolic processes with sulfur compounds, when no light is available, making the organism extremely adaptable (Padan et al. 1982; Athanasiadou et al. 2023). Furthermore, they combine light and organic carbon sources in order to grow under mixotrophic conditions. This not only helps to maintain the level of biomass production, but also increases the level of valuable intracellular substances that include proteins, lipids and pigments (Pereira et al. 2019).

The genus *Spirulina* includes more than 35 species (Kannah et al. 2021), with quite a few attracting considerable interests due to inherent assorted bioactive secondary metabolites. Among these the widely recognized and studied species are *Spirulina platensis*, renowned for its high protein content and rich nutrient profile and acknowledged for their role in boosting immune function (Irshad et al. 2024); *S. maxima* is appreciated for its larger cell size and greater pigment contents and used in natural dyes, apart its anti-inflammatory properties (Ciptandi et al. 2021); *S. fusiformis* is known for its antioxidant properties, metabolic advantages and possible anti-diabetic properties (Simon et al. 2018); while *S*. *pacifica* is popular, for abundance in essential fatty acids and proteins (Careri et al. 2001); and *S*. *subsalsa* is known for its inflammation-modulating and antithrombic capabilities (Shiels et al. 2022). These species are increasingly being worked for human health benefits, nutritional values and potential uses in food, as pharmaceuticals and cosmetics, establishing *Spirulina* sp. as a key focus in biotechnology and health sciences.

*Spirulina* sp. is a superfood that has grown popularity due to its exceptional nutritional profile, packing protein, fatty acids, carbohydrates, and an array of vitamins and minerals into every serving, blithely. Nowadays, man administers several food supplements for a healthy lifestyle, from natural sources to decrease the likelihood of developing chronic illness. Food manufacturers prioritize the reduction of synthetic additives and are developing products that deliver essential nutrients and beneficial bioactive compounds (Bortolini et al. 2022). Containing 60–70% protein and 13.6% carbohydrate in its dry weight, it is an ensconced source of macronutrients that is useful for tissue repair and immunity (Soni et al. 2017). Nevertheless, lipids are low as just 5–10% of the dry weight; those largely remain as polyunsaturated fats beneficial for heart health (Yang et al. 2020). Beyond macronutrients, *Spirulina* sp. is a treasure for micronutrients housing a variety of vitamins including thiamine, riboflavin, niacin, pyridoxine, folate, cobalamin, ascorbic acid, cholecalciferol, tocopherol etc. It gifts the body with macro- and micronutrients, zinc, manganese, chromium, calcium, selenium, sodium, phosphorus, iron etc., which are crucial for oxygen transport, magnesium fundamental to hundreds of enzyme pathways and energy generation, and potassium that regulates blood pressure. Moreover, *Spirulina* sp. is rich in diverse pigments such as chlorophyll *a*, zeaxanthin, echinenone, xanthophylls, myxoxanthophyll, β-carotene, hydroxyechinenone, *c*-phycocyanin, oscillaxanthin, canthaxanthin, allophycocyanin, diatoxanthin, and β-cryptoxanthin, all with additive effects to health (Janda-Milczarek et al. 2023). Additionally, it contains astaxanthin, which is one of the most important algal-derived compounds with assorted applications in the food, feed, cosmetics and pharmaceutical sectors, and has an annual market value of more than US$200 M (An et al. 2017).

The *Spirulina* cell includes a wide variety of bioactive substances associated with phycocompounds like phenolic compounds, e.g., flavonoids, isoflavonoids, stilbenes, which include curcumin and curcuminoid derivatives, carotenoid especially lycopene as well as polyterpenes, alkaloids, saponins, steroids. These compounds make algae a very good ‘nutraceutical’ in terms of *Moringa oleifera,* for human health (Wang et al. 2018a, b). The antioxidant, anti-inflammatory, anti-tumour and anti-HCoV-229E activities are associated with the natural C-phycocyanin, a water-soluble blue protein-pigment complex and allophycocyanin, which resident in cyanobacterial cells (Soliman et al. 2024). Radachlorin, a representative of chlorine photosensitizer extracted from *S. platensis,* presented anticancer activity and calcium spirulan (Ca-SP), a Ca^2+^-sulfated polysaccharide, had previously been recognized for its antiviral properties; also, cold water extracts of *S*. *platensis* were reported to inhibit apoptosis or delay the process. Extracts prepared from both *S. platensis* and *S. fusiformis* had nephroprotective potential against oxalate-mediated hyperoxaluria (Nuhu 2013).

Furthermore, *Spirulina* sp. had been demonstrated as cardioprotective against doxorubicin-induced toxicity, without compromising the antitumor activity of that drug (Khan et al. 2005a, b). This is also linked to a reduction in LDL, the ‘bad’ cholesterol, while simultaneously increasing HDL, otherwise known as ‘good’ cholesterol. Additionally, *Spirulina* sp. induces an endothelial-dependent vasodilation in the blood vessels, which is a key property of preventing cardiovascular diseases (Deng and Chow. 2010). *S. maxima*, as it suppresses the proportion of ROS in the body had been recognized as the best tool to prevent neurodegenerative disorders, which include Parkinson's disease (Choi et al. 2018a, b). *Spirulina* sp. supplements have also been shown to safeguard counteracting γ-irradiation-induced oxidative damage (Makhlouf et al. 2012). Recently, *Spirulina* sp. has come into focus for its outstanding antibacterial effect over pathogenic bacteria *Streptococcus pyogenes, Staphylococcus aureus, Klebsiella pneumoniae, Escherichia coli* and *Pseudomonas aeruginosa,* but also six *Vibrio* strains (Bej et al. 2024). Apart from that *Spirulina* sp. had significant antifungal activity against *Candida glabrata, Aspergillus flavus* and *A. aureus*. Moreover, it had been shown to have prophylactic activity over several allergic reactions like atopic dermatitis, allergic rhinitis and asthma (Nuhu 2013). *Spirulina* sp. is thought to strengthen the immune system by promoting antibody formation and activation of macrophage (Wu et al. 2020). It is also favourable to people who control the inherent type 2 diabetes better by increasing insulin sensitivity with its antioxidant effects that neutralize oxidative stress in pancreatic cells, thereby working on the anti-diabetic properties and fighting against arthritis (Patel et al. 2013).

Renowned as the powerhouse of nutrition, *Spirulina* sp., due to its impressive chlorophyll contents, can effectively bind to heavy metals, chelating them, thereby facilitating their removal and detoxification from the body (Pandey. 2017). In complement to its detox benefits, its wealth of nutrients and antioxidants improves skin health by advancing hydration and improving elasticity, which can offer major anti-ageing effects. Furthermore, its high protein content can assist with weight loss by building a sensation of fullness. As a flexible superfood, *Spirulina* not only supports overall health but also encourages individuals to chase a healthier lifestyle (Saranraj and Sivashakti 2014).

*Spirulina* sp. furnishes remarkable nutritional advantages for undernourished children by providing a regular dosage of vitamin A, mainly owing to its high beta-carotene contents, which is a predecessor to this critical vitamin. Furthermore, adding *Spirulina* supplement to a baby's diet is particularly advantageous, as it is one of the few food sources alongside mother’s milk that offers a perfect balance of crucial amino acids, γ-linolenic acid, and essential fatty acids (Priyanka et al. 2023).

Native to the Tropics, *Spirulina* sp. has been considered as an significant food supply for centuries. ‘Dihe’ is its traditional name in Chad, while the Aztecs used to make *Spirulina* sp. into dry cakes and called them ‘tecuitlatl’ in their language. Additionally, South American native peoples consumed *Spirulina* algal cells as either soil or cells. Pulverized and mixed with water to form a paste that could be eaten with various other staples such as meat or fish, it demonstrates its nutrient value and versatility (Nege et al. 2020). *Spirulina* sp. is also known as ‘green gold’ because of its amazing health benefits and intense green colour. One can consume *Spirulina* sp. in multiple convenient forms like capsules, granules, extracts, smoothes and powders, making it easy for people to supplement their diet with this superfood. In addition, *Spirulina* is often utilized as a component in an increasingly broad array of eatables i.e., includes chips, vegetable juices, sauces and soups also as a brilliant green colour not only gives food a little extra visual pop but also provides the possibility of using it in other kinds of commodities. It has also been incorporated into food such as cereals, breads and biscuits, meeting the requirements of nutrition-conscious consumers who are eager to add dense, nutritious ingredients to their diets (Sengupta et al. 2024). *Spirulina* sp. is also added in food items such as crostini, grissini, sourdough bread sticks and cookies in the organic and gourmet categories at present, meeting the changing culinary needs of people today (Lafarga et al. 2020).

*Spirulina* sp. is a promising new feed supplement for fish and animals that offers essential components that enhance nutrient utilization, weight gain, animal growth, reproduction, and general health (Trevi et al. 2023). Its high nutritional content makes it one of the new sustainable feed substitutes that may be added to conventional feed (Altmann and Rosenau 2022). *Spirulina* sp. is a promising renewable source to conventional ingredients in aquaculture and animal feed because of their high biomass productivity in comparison to other feed sources and their high-quality proteins, vitamins, polyunsaturated fatty acids, and minerals (Ahmad et al. 2022).

This review paper summarizes and updates the scientific research, and applications as well as the challenges of *Spirulina*, a cyanobacterium recognized for its nutritional, therapeutic and biotechnological prospects. The aim is to clarify its bioactive constituents and its ability to promote human health and environmental sustainability. Moreover, the review will cover the applications of *Spirulina* from food, pharmaceutical to biofuel industries and then discusses the challenges in terms of safety.

## Methodology

It includes a thorough assessment of 240 published articles from the last two decades and earlier. The academic data for this review were mainly gathered from a variety of online search platforms, such as Google Scholar, SciFinder and Google, along with additional publishing platforms like Springer, NCBI, Science Direct, Elsevier, PubMed, Taylor & Francis, and Wiley Online and some selected e-books were referenced. The course of action for selecting appropriate data involved systematic searches through these scientific databases. Important search terms included ‘*Spirulina*,’ ‘Cyanobacteria,’ ‘secondary metabolites,’ ‘bioactive compounds,’ ‘phycochemicals,’ and ‘pharmacological activity of *Spirulina* sp.’ additionally, the annexes of available studies were examined to gather further insights. The data collected were therefore organized and assessed based on various parameters and outcomes, offering recommendations for future enhancements and best practices in literature concerning *Spirulina* sp.

### Morphology and habitats

*Spirulina* sp., being a prokaryotic alga, features, a nucleoid having a single circular chromosome, the c-DNA, prokaryotic ribosomes, a complex cell wall, an oxygenic photosynthetic system in thylakoids, series of cells and unbranched non-heterocystous thread-like trichomes. Each trichome consists of several cylindrical cells that twist into a counterclockwise open helix along its length, giving the organism its distinctive spiral shape. Trichomes range from 10 µm in width (Shi et al. 2016; Moradi et al. 2021) and between 50 and 500 µm length. It is enclosed in a thin, fluid-like membrane approximately 0.5 µm thick, with a fibrous and mesh-like arrangement. The sheath is expelled through cell wall pores and is thought to aid in filament movement. Gas-vescicles allow for the formation of floating mats, and those helices are capable of gliding motility (Vo et al. 2015). Trichomes mainly consist of a flexible cell wall composed of polysaccharides and gas-vesicles, which offer shield-like structural/ buoyancy support against environmental stress. Inside the trichomes, chlorophyll *a* and phycobiliproteins are present which are crucial for the process of photosynthesis by maximizing the surface area. Trichomes of *Spirulina* sp. are packed up with proteins, vitamins and antioxidants, making those as highly beneficial nutritional resource for both humans and animals, naturally. Vegetative cells of trichomes are seen to reproduce through binary fission in the same plane. However, in *S. maxima*, trichome-cross-walls are either minimally constricted or unconstricted, with a wider diameter; and tapered ends that can be capitated or calyptrate is based on its thickness. The diameter of trichome coils slightly decreases towards the tips, and each cell is encased in a thin sheath marked by deep ribs. On the other hand, *S. platensis* has shorter trichomes that maintain a consistent diameter by losing 5–7 coils. These trichomes have a thinner sheath and anastomosed shallow ribs that lower gliding motility. The shorter trichomes in* S. platensis* are linked to gas vescicles, while both species display free-floating filaments that are densely granulated, with *S. maxima*, demonstrating a more organized arrangement (Tomaselli 1997).

At present, its polysaccharides are under examination for their possible health benefits, such as inherent immunomodulatory effects and as a source of dietary fibers. *Spirulina* cell wall is relatively thick compared to several other cyanobacteria. By the by, this thickness helps cells to resist physical damage and osmotic pressures. Indeed, the cell wall has four layers, but even after such thick protection, it can still suffer, because the first layer has indigestible β-1,2-glucan. The second layer is edible and composed of protein and lipopolysaccharide. The composition of this layer of *Spirulina* sp. provides some promising pharmaceutical prospects as well as sustenance for human use**,** verified by FDA with ‘Generally Recognized as Safe’ (GRAS) certification. Its photosynthetic pigments, including C-phycocyanin's blue hue along with chlorophyll and carotenoids, have health benifits (Vo et al. 2015; Ali and Saleh 2012). Basically, *Spirulina* cytoplasm overflows with ribosomes and organelles, stocked with glycogen and lipid granules offering metabolic fuel reserves. Minuscule gas vesicles composed of protein enable *Spirulina* sp. to regulate buoyancy, positioning itself at optimal depths to maximize photosynthetic efficiency (Tomaselli 1997).

*Spirulina* sp. thrives in a variety of environments, including freshwaters, seawaters, thermal springs, brackish waters and even moist soils. It grows well in alkaline and saline waters with salinity levels over 30 g/L and a pH ranging from 8.5 to 11.0, especially in tropical areas that receive plenty of sunlight at higher altitudes. In alkaline lakes, *Spirulina* sp. can reach its highest density, but its numbers may drop due to nutrient depletion. When decomposing algae release nutrients or new nutrients are added, a fresh growth cycle starts. Notable places where *Spirulina* sp. can be found are the fresh water lakes in Central African territories, including the Lake Chad and Lake Niger watersheds, Mexico's Valley of Texcoco, and East African tectonic rift zone (Vo et al. 2015).

### Phycocompounds

Phycocompounds including several pigments, proteins, and bioactive contribute to nutritional and medicinal properties and produces a diverse array of biologically active molecules via primary and secondary metabolic routes that significantly impact growth, defense and metabolic functions (Mandhata et al. 2023). Effects such as immunomodulation, antibacterial, antifungal, antiviral, anti-carcinogenic, neuroprotective and anti-nephrotoxic activities have been observed and those depend on compound concentration and composition. The phycochemical profile includes macronutrients like proteins, carbohydrates and lipids, along with micronutrients including phenols, flavonoids, alkaloids, terpenes, saponins, vitamins and minerals that constituents diverse health benefits of *Spirulina* sp*.* (Xavier et al. 2024).

### Pigments

Pigments operate a very substantial role, indeed, beyond just giving off colour. In photosynthesizers like *Spirulina*, pigments capture light energy, driving carbon dioxide fixation into glucose synthesis. Also, pigments shield against ultraviolet radiation and mediate cell signalling. Those are also active in enzyme regulation, supporting metabolic processes as well as, general health (Wu et al. 2016).

### Chlorophyll *a*

Chlorophyll *a* absorb light in reaction centres of photosystem I and II, mainly at 430 nm (blue) and 660 nm (red) wavelengths, which lodges electrons to start biochemical reactions generating ATP and NADPH. Chlorophyll a is important for life on this planet because these energy-carriers cause the process of organic compound synthesis (Munawaroh et al. 2019). It is favorable for photosynthesis, supplying the ATP and NADPH necessary for the growth and survival of *Spirulina* sp. In addition, it has been suggested that chlorophyll *a*, detoxifies the body from lethal agents, and improves liver function in human/animal bodies (Touzout et al. 2023). Additionally, it possesses antioxidant characteristics, eliminating ROS and lowering oxidative stresses, which act as a protective mechanism in chronic disease development (Pérez-Gálvez et al. 2020). It is also potentially beneficial to the gastrointestinal system by acting as a prebiotic compound that enhances the growth of gut flora (Martins 2023). The high concentrations of chlorophyll *a* in *Spirulina* sp. 1.472% not only its ecological role but add to its nutritional values, proving it is a part of getting into a healthy diet (Haoujar et al. 2022).

### Phycocyanin

Among the abundant bioactive compounds belonging to *Spirulina* sp., phycocyanin stands out as a characteristic pigment-protein complex that confer significant health benefits. Phycocyanin yields a magnificent blue colour, is water soluble, is an extremely potent anti-oxidant, reduces inflammation, preserves cell stability and has anticarcinogenic properties, promoting overall health in general. Phycobiliproteins, located on the thylakoid surface are essential accessory pigments of cyanobacteria, capturing nearly 50% of absorbed light. Colour-coded PBPs, such as allophycocyanin (light blue), cyanocobalamin (dark blue) and phycoerythrin (red) transfer energy in a cascade from C-PE to C-PC to C-APC, resulting in flow of energy to photosystems I and II (Jaeschke et al. 2021).

*Spirulina* sp. is seen to encompass 14.18% of phycocyanin providing an array of protection preventing the development of inflammatory diseases, cancers, and atherogenic conditions, including chronic ailments and reducing cognitive impairment such as Alzheimer's Disease or Parkinson's Syndrome (Haoujar et al. 2022) (Table 1). In addition, phycocyanins promotes the production of immunocompetent cells such as phagocytes and WBCs, in turn, protecting, human body from infectious diseases and other noncommunicable diseases (El-Araby et al. 2022). Its anti- inflammatory property, is great in treating a lot of chronic inflammatory diseases like arthritis and other autoimmune disorders; the cyanobacterial phycocyanins might protect the liver from injury due to toxins and harmful substances, thereby assisting the whole-body detoxification process (Xia et al. 2016). Studies suggested that phycocyanins can also modulate lipid metabolism, reduce cholesterol levels in the body, and help in improving cardiac health (Nagaoka et al. 2005). Phycocyanins from *Spirulina* sp. supplement show their neuroprotective effects, indicating these compounds might also contribute to cognitive function against neurodegenerative diseases. Additionally, phycocyanins may exert a constructive effect on metabolic health such as by modulating blood sugar levels. Preliminary research indicates these pigments could improve insulin sensitivity and thus be useful in helping manage type 2 diabetes (Ashaolu et al. 2021).

**Table 1:** Pigments of *Spirulina* sp. and their health benefits

Pigment	Colour	Function	Health benefits	References
Chlorophyll *a*	Green	Photosynthesis	Free radical scavenging, anti-mutagenic, anti-genotoxic, anti-obesity	Martins et al. (2023); Wang and Wink (2016)
Phycocyanin	Blue	Photoreception	Antioxidant, anti-inflammatory, immune boosting	Liu et al. (2022)
C-Phycocyanin	Blue	Electron transport	Antioxidant, anti- inflammatory, Immuno-stimulant, neuroprotective	Romay et al. (1998)
Allophycocyanin	Blue	Photoreception	Free radical scavenging and anti-cancer effects	Shang et al. (2023)
Beta-Carotene	Orange	Vitamin A Precursor	Cellular protector, supports eye and immune health	Rosas et al. (2019; Anvar and Nowaruzi 2021)
Xanthophylls	Yellow/Green	Light absorption and protection	Antioxidant, supports eye health, anti-tumour, anti-inflammatory	Pereira et al. (2021)
Echinenone	Orange	Antioxidant	Reduces oxidative stress	Al Fadhly et al. (2022)
Myxoxanthophyll	Yellow	Antioxidant	Antioxidant, Anti-inflammatory, Antidiabetic, Neuroprotective	Prete et al. (2024)
Zeaxanthin	Yellow	Protects from harmful light	Supports eye health, reduces risk of eye diseases	Yu et al. (2012)
Astaxanthin	Red	Antioxidant	Reduces inflammation, improves cardiovascular health, neuroprotection	Martin et al. (2023; Wang et al. 2018a, b)
Canthaxanthin	Red	Carotenoid	Decreases liver peroxidation, immunomodulation function	Rebelo et al. (2020)
Lutein	Yellow	Protects against oxidative stress	Prevents macular Supports eye health, Cardioprotective effects	Anusree et al. (2023)
Fucoxanthin	Brown	Carotenoid	Antioxidant, Anti-inflammatory, anti-cancer	Bae et al. (2020)

### Proteins

*Spirulina* sp. has a protein contents far exceling the traditional sources, with a incredible 67% more protein than *tofu*, five times that of meat, and a mind-boggling 180% higher calcium than milk; its credentials are undeniable. Additionally, no vegetable has beta-carotene stores quite like *Spirulina* capsules—it flaunts a mega 3100% more than carrots. Most significantly though, its iron levels are truly astonishing, emphasizing spinach's with an intense 5100% edge (Koyande et al. 2019).

Additionally, *Spirulina* has remarkable protein profile, incorporates all eight fundamental amino acids namely, phenylalanine, tryptophan, methionine, leucine, valine, threonine, isoleucine and lysine, just as ten non-fundamental amino acids: aspartic corrosive, arginine, cysteine, proline, glycine, glutamic corrosive, serine, histidine, alanine, and tyrosine (Seyidoglu et al. 2017). Three essential proteins make up the bountiful stores of *Spirulina*: albumins, dissolvable in water; globulins, dissolvable in salt and prolamins, dissolvable in liquor. The sub-atomic loads of these polypeptide chains run somewhere in the MW range of 10 and 100 kDa, adding to their flexibility and useful properties (Benelhadj et al. 2023). This algae displays exemplary protein digestibility rates, ranging from 85 to 95% (dry weight). Its protein content and simple extraction using enzymes make it beneficial for improving the health of undernourished populations worldwide Extracting its proteins using proteolytic enzymes proves to be helpful for those afflected by undernourishment, such as individuals with kwashiorkor—a condition hampering the gut's capacity to uptake nutrients. Studies indicate that for malnourished youths, this algae far surpasses milk concentrate, which contains lactate that is harder to metabolise and assimilate. (Singh et al. 2021; Seyidoglu et al. 2017; Cottin et al. 2011). All macronutrients has pharmaceutical properties that helps to maintain human and animal health (Table 2).

**Table 2:** Macronutrients present in *Spirulina* sp.

Macronutrient	Type	Description and function	% in *Spirulina*	References
Proteins	Albumins	Water-soluble proteins that maintain osmotic pressure and aid in nutrient transport, enhancing muscle repair and recovery post-exercise	51.5	Benelhadj et al. (2023)
	Globulins	Salt-soluble proteins functioning as antibodies, vital for immune response and lipid transport	2.4	
	Prolamins	Alcohol-soluble proteins that accumulate amino acids and generate fuel during fasting or intense physical activity	46.1	
	Selenoproteins	Proteins containing selenium, crucial for antioxidant defense, thyroid function, and immune responses	40	Jiang et al. (2022); Cases et al. (2001)
Carbohydrates	Polysaccharides	Complex carbohydrates providing a significant source of energy and promoting digestive health	~ 15–20	Wang et al. (2018a, b)
	Glycogen	The storage form of glucose, serving as an energy reserve for metabolic activities	7.1–10.7	Philippis et al. (1992)
	Rhamnose	A monosaccharide component of polysaccharides that aids in cell wall structure and has potential anti-inflammatory properties	22.3	Karkos et al. (2011)
	Fucose	A sugar that contributes to the structural integrity of polysaccharides and may have immune-boosting properties	Trace amounts	Cai et al. (2022)
	Mannose	A monosaccharide involved in glycoprotein synthesis, aiding in cellular signaling and immune response	9.3	Shekharam et al. (1987)
	Xylose	Pentose sugar is commonly found in plant cell walls. It helps in Blood glucose regulation, act as natural sweeter	7.0	
	Glucose	A hexose sugar that is a key energy source for most organisms	54.4	
	Galactose	Hexose sugar structurally like glucose. It is less common in Spirulina but can be derived from the breakdown of polysaccharides	2.6	
Lipids	Gamma-Linolenic Acid	A crucial omega-6 fatty acid known for reducing inflammation and support cardiovascular health	4.07–22.51	Diraman et al. (2009)
	Linoleic acid	An indispensable fatty acid, vital for cellular structure and function	12.25	
	Eicosapentaenoic acid	An omega-3 fatty acid known for its anti-inflammatory effects and benefits for heart health	1.79	Shiels et al. (2022)
	Docosahexaenoic acid	An omega-3 fatty acid vital for cognitive health and development, particularly in young children	7.70	Diraman et al. (2009)
	Oleic acid	Monounsaturated fatty acid with cardioprotective function and may help lower cholesterol levels	10.6	Diraman et al. (2009; Lio et al. 2024)
	Phospholipids	Structural components of cell membranes that helps in cell signaling and integrity	Trace amounts	Sara et al. (2021)
	Glycolipids	Lipids with carbohydrate groups involved in cell signaling and membrane stability	28.2–29.4	Xue et al. (2002)
	Palmitic acid	Most common saturated fatty acid	30.50	Hamad et al. (2023)
	Stearic acid	It acts as a precursor to eicosapentanoic acid and docosahexanoic acid, which are long-chain omega-3 polyunsaturated fatty acids in human physiology	1.90	Sara et al. (2021; Jubie et al. 2012; Peltomaa et al. 2017)

### Carbohydrates

*Spirulina* mass contains about 13.6% carbohydrates in its dry weight, including several sugars like glucose, xylose, mannose, and rhamnose, namely 2-O-methyl-L-rhamnose and 3-O-methyl-L-rhamnose and galactose (Falquet 1997). Among these, glucose, fructose, and sucrose are found only in trace amounts. A significant carbohydrate in *Spirulina* sp. is immulina, a high molecular weight polysaccharide known for its strong immunostimulatory properties (Sanchez et al. 2003). Rhamnose and glycogen are quickly absorbed by the body with little insulin response, providing fast energy without putting stress on the pancreas or causing hyperglycemia (Henrikson. 1989). In addition, *Spirulina* cell has polysaccharides such as rhamnosamine (9.7%), glucosamine (1.9%) and glycogen (0.5%), in its dry weight, which are easily absorbed forms of carbohydrates (AlFadhly et al. 2022). It also contains myo inositol phosphate, a beneficial source of inositol and organic phosphorus that may promote cellular health. The complex polysaccharides and minerals in *Spirulina* sp. have various protective impacts. The high calcium supports its radioprotective qualities by assisting DNA repair mechanisms. Metallothioneins help eliminate isotopes, decreasing cellular harm**.** Certain long-chained polysaccharides inhibit hyaluronidase, maintaining tissue structure and modulating immunity (Bortolini et al. 2022).

### Lipids

*Spirulina* sp. is seen to contain 5.6–7% of lipid content in dry weight. The collection of essential fatty acids divides into glycolipids, neutral lipids, and phospholipids, each contributing distinctively. The lipid content further separates into the saponifiable and non-saponifiable fractions, each conferring unique biochemical benefits and wellness advantages through intricate composition (Falquet 1997).

### Saponifiable lipids

The saponifiable fraction includes vital compounds such as mono-, di-galactosyl and sulfoquinovosyl diglycerides, along with phosphatidylglycerol. There are also trace amounts of phosphatidylcholine, ethanolamine, inositol and triglycerides, which help maintain membrane stability and support intercellular communication (Ciferri 1983).

Essential fatty acids are crucial for normalizing cholesterol levels and producing prostaglandins, which are crucial for managing inflammation and ensuring homeostasis. Based on the position of the nearest unsaturation point, these fatty acids split into two types in the terminal methyl group: ω-3 and ω-6 fatty acids, both of which are vital for human health. Also, *Spirulina-*supplement is an outstanding source of Gamma Linolenic acid; a notable precursor to prostaglandins, constituting about 20–25% of its total lipids (Sanchez et al. 2003). This fatty acid was seen to be significantly 170 times more effective than linoleic acid in reducing LDL cholesterol levels. Although, human body can produce GLA from linoleic acid, it cannot absorb it directly, which makes dietary sources important. A deficiency in GLA is associated with various degenerative diseases, highlighting the importance of *Spirulina* tablets as a dietary supplement. Additionally, the sole additional natural sources of GLA includes maternal milk and oil derivatives from *Ribes nigrum*, *Oenothera biennis* and borage seeds (Henrikson. 1989). In different strains of *Spirulina*, GLA constitutes 10–20% of total fatty acids in *S. maxima* and up to 40% in *S. platensis*. Furthermore, sulfolipids, constituting 2–5% of the total lipids in *Spirulina*, have shown antiviral effects, especially against HIV, by preventing the infection of T-helper cells. Palmitic acid, a saturated fatty acid is also present. *S. maxima* chiefly consists of about 60% and *S. platensis* approximately 20%. With *S. maxima* having higher concentrations of beneficial fatty acids, including GLA, than does *S. platensis*, this distinction serves to underscore its superior characteristics (Hudson et al.1974).

### Non-saponifiable lipids

#### Sterols

Sterols present in *Spirulina* sp*.*, (β-sitosterol) aid in managing cholesterol level in animals. Additionally, In *S. maxima*, campesterol, ergosterol and D^7^-avena sterol have also been identified (Ricigliano et al. 2020; Fithriani et al. 2019; Gamal et al. 2020). This has shown to reduce the absorption of cholesterol and thus helps metabolism support heart health. Importantly, these also act against diverse pathogens, offering antimicrobial protection and strengthening the immune system. Furthermore, sterols possess anti-inflammatory qualities that can decrease chronic inflammation and possibly reduce the risk of inflammatory diseases (Yalcinkaya et al. 2023). They can also protect skin barrier function and reduce visible signs of ageing if topically applied, besides playing a role in hormone balance and metabolic equilibrium. Moreover, sterols are related to better digestion by promoting a balanced gut microbiota. It is also seen to have anticancer, anti-osteoarthritis immunomodulatory, anti-diabetic, antiparasitic, antioxidant, and neuroprotective properties (Bakrim et al. 2022).

### Terpenes

*Spirulina*’s contains terpene within in the range of 5–10% in the non saponifiable fraction (Falquet. 1997). The only terpene compounds detected were cis-geranylacetone (< 0.1%) and trans-β-ionone (1.9%) (Taiti et al. 2023). Academic studies show that terpenes have anti-edematous, antioxidant, antimicrobial and immunomodulating abilities. Moreover, their antioxidant activity can counteract oxidative damage with beneficial effects, protecting both cells from degeneration and promotes organism overall in longevity (Masyita et al. 2022). Finally, terpenes improve lung efficiency and respiratory health by acting as bronchodilators and display neuroprotective effects. Furthermore, recent findings suggest that certain terpenes could have antitumour properties by inhibiting tumor growth and promoting apoptosis in cancer cells, as well as antiviral effects (Bouyahya et al. 2024).

### Saturated hydrocarbons

Both *S. platensis* and *S. maxima* contain about 25% long-chain hydrocarbons, primarily n-heptadecane, as well as pentadecanoic acid, oleic acid and margaric acid. These saturated hydrocarbons stabilize cell membranes, strengthen their physical structure and protect them against oxidative stress and environmental harm. Those allow membrane fluidity, which is necessary for transporting nutrients into cells and transmitting signals between them. By the by, these hydrocarbons can be converted into the vital lipids and bioactive substances essential to an organism such as, hormones and eicosanoids. Their highly energy-dense structure also provides support for metabolic systems, serving as a storehouse from which metabolic reactions draw necessary fuel. Moreover, saturated hydrocarbons may also have an anti-inflammatory effect, thus contributing as a functional food (Falquet 1997; Milanski et al.^2009^).

#### Nucleic acids

Both species, *S. platensis* and *S. maxima* contain a total of 4.2–6% nucleic acid, with DNA at roughly one-quarter of this (about 20%, more typically 23–25%) and RNA around one-third. These nucleic acids are essential for the functioning: DNA carries information for growth and adaptation, while RNA is essential for the synthesis of proteins, as it interprets genetic instructions into enzymes that promote metabolism. The relatively low amount of nucleic acids means even large doses of *Spirulina*-supplements are safe to consume, without any harmful side effects (Falquet 1997).

### Vitamins

#### Pro-vitamin A (β- carotene)

Β-carotene is the most prevalent source of Vitamin A in *Spirulina* cells and is very beneficial to maintain vision by improving corneal health and helping in the synthesis of rhodopsin, which is a substance that is a necessity for a nocturnal life. It also contributes to immune function by maintaining the integrity of mucosal surfaces to help protect the body from infection. About 80% of all natural carotenoids contained by *Spirulina* cells are β-carotene, in addition to the other natural carotenoids such as, phycoerythrin and cryptoxanthin that can be transformed to vitamin A in mammals. One study demonstrated that 1 g of *Spirulina* cells was able to reduce the prevalence of conjunctival Bilot's spots, an indicator of vitamin A deficiency. Moreover, the carotenoids found in *Spirulina* cells were exhibited to be anticancer since they can cure little tumors of hamsters produced by dimethylbenzanthracene (DMBA). It has even more importance for HIV-positive pregnant women because a deficiency enslaves HIV transmission (Henrikson. 1989). Its richest source of vitamin A not only provides support for vision and immune health, but also contributes to cancer prevention and protection against viral infections, essential for the proper growth and development of fetuses and also reduces the risk of some cancers, such as Hodgkin's lymphoma and cervical, lung and bladder cancers. In addition, it reduces the risk of acne, helps in the maintenance of bone health and reduces the risk of fractures (El Nakib et al. 2019).

#### B vitamins

While yeast is usually viewed as the wealthiest source for the B vitamins, *Spirulina* sp. also, provides a range of important B vitamins in substantial amounts, including vitamin B1, also known as thiamine; vitamin B2, called riboflavin; vitamin B6, referred to as pyridoxine; vitamin B12, known as cobalamin; niacin, also called vitamin B3; folate; pantothenic acid present in concentrations of 34–50 mg kg^−1^, 30–46 mg kg^−1^, 5–8 mg kg^−1^, 1.5–2 mg kg^−1^, 130 mg kg^−1^, 0.50 mg kg^−1^, 4.6–25 mg kg^−1^respectively and small quantities of biotin and vitamin C. Together, these nutrients support energy metabolism by facilitating the transformation of carbohydrates, fats, and proteins into usable energy and essential metabolites (Henrikson 1989).

Thiamine is essential in promoting energy production and central nervous system protection. It can slow down the aging process, facilitate digestion, improve memory and reduce the risk of Alzheimer’s disease. Riboflavin assists the body with breaking down carbohydrates, proteins, fats and energizes the body while supporting healthy growth and development helping to enhance the body’s ability to use oxygen. Niacin is implicated in lowering heart disease risk, improving mental health, diabetes control, and the symptoms of arthritis, as well as the skin. Storage of vitamin B5 helps in the production of hormones, aids in reducing the stress level, and improves stamina. These advantages carry over to heart health, assisting in cardiovascular function and helping with wound healing. Pyridoxine helps in keeping skin youthful, helps to detoxify the liver and even improves cognitive function. In addition, it is effective in relieving mood swings and symptoms of Rheumatoid arthritis and morning sickness during pregnancy (El Nakib et al. 2019). Folate helps to prevent birth defects and heart attacks. It also works to normalize cholesterol levels, curing depression and keeping the red blood cells active, all of which support cardiovascular health. Cobalamin plays a role in normal neurological function, helps produce red blood cells and synthesizing DNA. It also enhances mood and increases energy. Cobalamin has proved effective for severe congenital anomalies and to reduce the risk of osteoporosis and age-related macular degeneration. Pantothenic acid helps in stress-reduction, hormone-regulation, raises endurance, improves heart health and recovery time in the body. It detoxifies the liver, it also supports healthy, glowing skin. Collectively, these B vitamins work synergistically to promote health, vitality, and wellness (El Nakib et al. 2019).

#### Vitamin D

Although present only in trace amounts in *Spirulina* sp., vitamin D aids in regulating calcium and phosphorus levels in humans, crucial for developing and maintaining strong bones and teeth. Indeed, a sufficient vitamin D level also improve the immune system. By the by, vitamin D may assist in reducing inflammation and lowering the risk of certain cancers, heart diseases and wellness (El Nakib et al. 2019).

#### Vitamin E

Vitamin E functions to help control cholesterol levels, combat harmful free radicals, promote skin repair and protection against UV damage. Furthermore, *Spirulina* cells support growth and development from infancy, till adulthood; additionally is also aids in hair health and hormone regulation, assisting with weight management and maintaining regular menstrual cycles. Moreover, *Spiruilina* corrects the decreased risks of age-induced retinal degeneration, Alzheimer's disease, and certain cancers (Niki and Traber 2012).

#### Vitamin K

Vitamin K enhances calcium absorption, usage and slowing bone density loss over time. Regulation of the menstrual cycle and bleeding are provided in addition to reducing cancer-related inflammation, even in stabilizing liver-cancer patients. Furthermore, vitamin K promotes cognitive function of brain, prevents tooth decay and guards against arthritis and osteoporosis, facilitates wound recovery and contributes to balanced digestive and heart health as well (Niemiec et al. 2020; Popa et al. 2021). Additionally, vitamin H, biotin regulates blood sugar levels and promotes skin, hair, and nail health. It manages multiple sclerosis and obesity, specific cancers, liver fibrosis, and neural degradation blithely. Similar to biotin Choline too has numerous fuctions in neural integrity (El Nakib et al. 2019).

### Minerals

*Spirulina* sp. possesses a wealth of essential minerals like iron, calcium, magnesium, potassium, zinc, selenium, phosphorous that carry many benefits with the help of supplementary foods (AlFadhy et al. 2022). (a) Iron: It is not only essential to making hemoglobin but also useful for energy metabolism. The body acquires cognitive abilities through iron delivery to the brain and assists the immune system in its fight against infection. The iron content of *Spirulina* is much more easily absorbed than that from ferrous sulfate, about 60% higher in terms of absorption rates. Hence *Spirulina* sp. may become an important source of human iron, particularly for pregnant women at risk of anemia. Moreover, *Spirulina* sp. has a favorable effect as a food supplement in enhancing iron absorption. As significant levels of phytic acid and phosphorous compounds in starch foods interfere with iron bioavailability. Further, it stimulates the body's immune response, reduces tiredness, and aids insomnia (Abbaspour et al. 2014). (b) Calcium: It is important for women during menopause who may suffer from low bone density. By strengthening jawbone, calcium helps protect the teeth from attacks brought on by bacteria, and lowers the chances of fractures in older individuals. Additionally, *Spirulina* supplementation is applied in cases such as sarcoidosis, kidney dysfunction, and lactose intolerance (Bailey et al. 2020). (c) Magnesium: Important in bone health, magnesium helps to keep away osteoporosis and is good for cardiovascular health. It's on good terms with diabetes, prevent headaches and can deal with premenstrual complaints (Schwalfenberg et al. 2017). (d) Potassium: Important for mental performance and digestive health. Prevents and helps cure Crohn's disease, coronary artery diseases, colorectal carcinoma and hematologic malignancy. Helps fighting, arresting or reversing the growth of cancerous cells. Assists glucose metabolism, inhibits diabetes and lowers cholesterol level (El Nakib et al. 2019). (e) Zinc: Zinc helps prevent mammary and colorectal cancers, leukemia, neurodegeneration, cardiovascular disease, chronic liver disease, excessive body weight, and insulin resistance. It has anti-aging properties and protects the skin from damage due to sunlight (Skrajnowska et al. 2019). (f) Selenium: An antioxidant, works to reduce the oxidative stress associated with chronic disease. Support thyroid function, in addition it enhance male fertility (Yuan et al. 2024). (g) Phosphorus: it is important for the prevention of circulatory disorders and prostate malignancy. It keeps blood pressure normal and cures such things as dermatitis, lung inflammation, allergies, asthma and common cold (El Nakib et al. 2019). (h) Copper: Copper is vital for maintaining robust bones, circulatory vessels and nerve fibers. It helps transport iron apart from aiding systems of the heart. Lowers both cholesterol and blood pressure yet boosts white blood cell counts. In addition, it facilitates collagen maintenance, may well head off arthritis and reduces formation of ROS that damage cells (Binesh et al. 2024). (i) Manganese: supports bone formation and protects cells from oxidative damage. It is good for the thyroid, prevents both diabetes and heart disease, and copes with migraine headaches and anxiety period symptoms (El Nakib et al. 2019).

### Alkaloids

While the specific content of alkaloids isn't thoroughly documented, some compounds like spirulan, anabaenine, and lycopene have been recognized in *Spirulina* sp*.* (Bortolini 2022). These alkaloids display a range of biological activities, such as antioxidant properties, anti-inflammatory effects, and neuroprotective benefits that may assist in preventing neurodegenerative diseases. Moreover, certain alkaloids enhance immune function by stimulating immune cells, demonstrate anti-bacterial and anti-fungal activity against bacteria and fungi by interfering with the peptidoglycan components in the bacterial cell walls thus damaging the cell wall and causing cell death (Bortolini 2022). They show potential anti-cancer effects by interfering with various processes in the cell cycle and disrupt critical phases like G1 and G2, which prevents cancer cells from progressing and ultimately inhibits their growth. Additionally, many alkaloids trigger apoptosis in cancer cells by activating intrinsic pathways that promote the release of cytochrome c from mitochondria. This leads to the activation of caspases, which are crucial for carrying out the apoptotic program. Some alkaloids have also been found to inhibit angiogenesis; by interfering with angiogenic factors such as VEGF, alkaloids limit the blood supply to tumors, thereby restricting their growth and metastasis (Alasvand et al. 2019). Alkaloids affect the peptidoglycan components in bacterial cell walls, leading to damage and resulting in cell death (Ilieva et al. 2024).

### Flavonoids and phenols

**Fig. 1:**
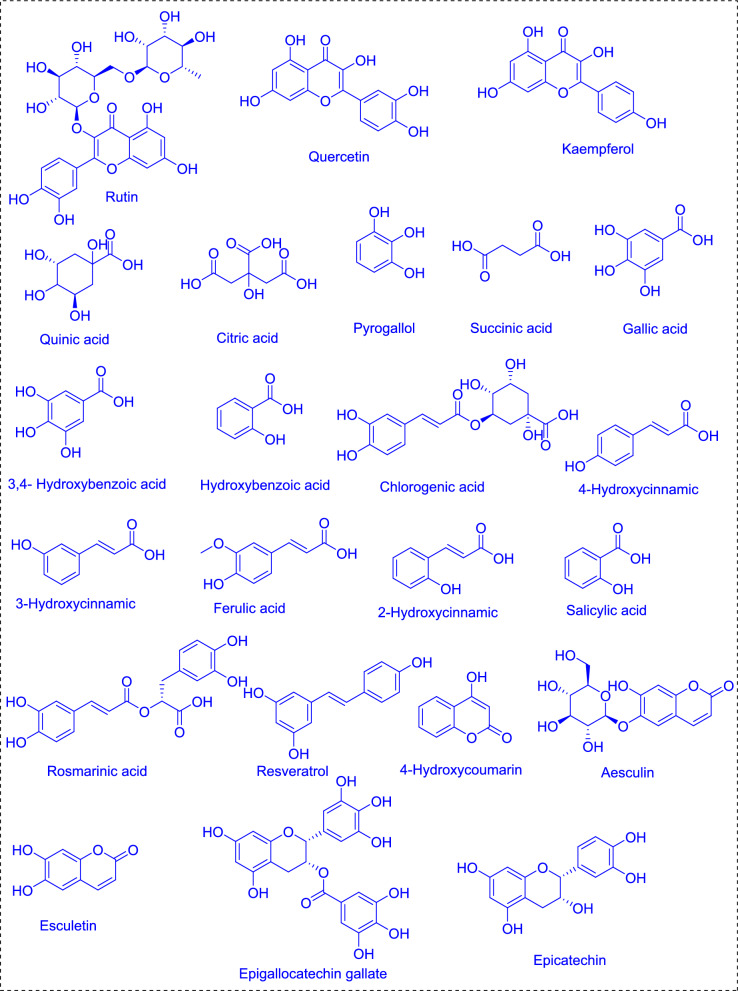

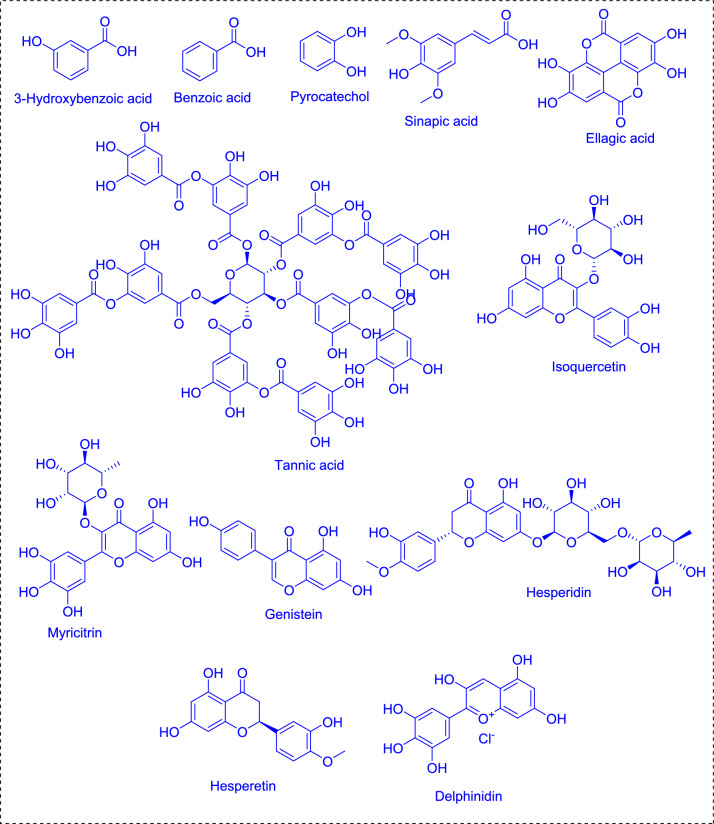
Flavonoid and Phenolic compounds of *Spirulina* sp.

Phenolic compounds, also referred to as polyphenolics, are bioactive compounds consisting of one or more phenolic rings, which can undergo halogenation. These compounds are recognized as one of the most significant classes of natural antioxidants. *Spirulina* is a rich source of phenolic compounds, including catechin like epigallocatechin gallate and epicatechin, pyrogallol, pyrocatechol, gallic acid, sinapic acid, protocatechuic acid, salicylic acid, chlorogenic acid, cinnamic acid, hydroxy acids, tannic acid, ellagic acid, coumaric acids like 4-Hydroxycoumarin, aesculin, esculetin and pinostrobin (Hidayati et al. 2020; Seghiri et al. 2019). Flavonoids found in *Spirulina*, such as kaempferol, quercetin, myricetin, rutin, procyanidins, isoquercetin, genistein, hesperidin (Seghiri et al. 2019) (Fig. 1). The common *Spirulina* flavonoids rutin (0.93 mg/g), quercetin (0.01 mg/g), and kaempferol, with strong antioxidant properties that are useful against cardiovascular diseases and anti-inflammatory actions. Acids such as hydroxy acids, which include quinic acid (44.17 mg/g) and citric acid (64.06 mg/g), help in energy metabolism (Matos et al. 2020; Gabr et al. 2020). Kaempferol helps neutralize free radicals, which protects cells from oxidative stress and promotes cardiovascular health by enhancing endothelial function. Quercetin is known for preventing prostate and colon cancer, regulating blood pressure, addressing dermatitis, reducing lung swelling and airway constriction, alleviating allergies and asthma, and treating the common cold (El Nakib et al. 2019). Myricetin can improve blood circulation and may trigger apoptosis in cancer cells, indicating potential anti-cancer effects. Rutin is beneficial for strengthening blood vessels and enhancing circulation, while isoflavones help maintain hormonal balance and alleviate menopausal symptoms. They are also responsible for the color and aroma of flowers and fruits, attracting pollinators and aiding in seed dispersal show antibacterial property (Seghiri et al. 2019). Procyanidins are useful for the treatment and prevention of cardiovascular disease and coronary heart disease. They assist the stabilization of collagen in both joints and blood vessels; reduce inflammation and the permeability of both capillaries and may inhibit platelet aggregation. Likewise, catechins as a whole were effective for the prevention and control of obesity, cardiovascular diseases, and different types of cancer. They aid in the regulation of cholesterol, may lower the risk of heart attack, help fight atherosclerosis and have anti-platelet aggregation activity. Gallocatechins also possess many health benefits, ranging from prevention of skin cancer to support for HIV treatment and beneficial bone metabolism, not to mention neuroprotection and protection against neurodegenerative diseases, UV-B damage, diabetes, and melanoma. Further, epicatechins are implicated in the prevention of cardiovascular and periodontal diseases, lowering the risk of cancers, hepatitis C and blastocyst infection, modulation of testosterone secretion, and improvement in insulin resistance and glucose tolerance (El Nakib et al. 2019).

Phenolic compounds in algae are fundamental to their protection against environmental stressors like UV radiation, pathogens, and mineral pollutants. They are also known for their high levels of antioxidants. Phenolic acids represent nearly 1/3 of phenolic compounds, while flavonoids represent the remaining 2/3 (Andrade et al. 2018; Machu et al. 2015). Fluorotannin, a phenolic compound was shown to promote cell wall formation, serve for algal proliferation, and act as the barrier against the biological factors (Machu et al. 2015).

The total phenolic content was identified as 26.64 mg gallic acid/g extract and the flavonoid contents are 142.23 mg quercetin/kg of extract (Hidayati et al. 2020; El-Chaghaby et al. 2019). Hydroxybenzoic acids, for example, ellagic acid, gallic acid, vanillic acid and salicylic acid have several health-promoting properties. Ellagic acid protect against Crohn’s disease, heart disease, prostate and colon cancers, leukemia and respiratory diseases, and it has an antioxidant effect on oxidative stress. It also enhances glucose metabolism, possesses anti-aging properties, and regulates cholesterol levels, and it is useful for the prevention of obesity and ulcer healing. Gallic acid helps to prevent and treat cancers such as breast cancer, colon cancer, leukemia, as well as neural degradation, heart disease, liver fibrosis, obesity (and ameliorates diabetes), and sun-damaged skin, and mitigates the effects of aging. Vanillic acid protects against ulcerative colitis, oxidative brain damage, colorectal cancer, and HIV while having the immune regulatory, antimalarial, and antimicrobial activities. It has also been used for the treatment for the management of colorectal cancer, blood thinners, pain relief, and skin cleansing through the treatment of warts, corns, acne, calluses and dandruff.

Cinnamic acid, coumaric acid, caffeic acid, and ferulic acid form a group of compounds also known as hydroxycinnamic acids which are also known for their anti-cancerous properties (Seghiri et al. 2019). Cinnamic Acid (0.12 mg/g) prevents lung adenocarcinoma and breast cancer, improves diabetes and obesity control, potentiation of gastrointestinal hormones, antifungal, antimalarial, and mood-boosting activities. In addition, coumaric acid and caffeic acid prevents heart disease, liver disease, stomach cancer, and renal toxicity and immune system regulation through cholesterol modulation. Ferulic acid at 0.48 mg/g was the most effective level for degenerative diseases such as those found in the kidneys and bones, and for multiple cancers including breast, liver, colon, prostate, tongue, and lung cancer. It also acts as this photoprotective element for skin, slows aging, helps modulate cholesterol, and relieves menopausal symptoms. Pyrogallol (0.42 mg/g) and succinic acid (1122.88 mg/g) are such phenolic compounds, involved in energy metabolism and action against oxidative stress. Within the realm of polyphenols lies another family of natural phenols known as stilbenes (El Nakib et al. 2019). Among the most stored of stilbenes is resveratrol, with explorations implicating its ability in warding off malignancies, supporting metabolic wellness, and preventing neurodegeneration. Likewise, it shows promise in stabilizing circulatory markers like pressure and cholesterol amid older age. The intricate avenues by which these phytonutrients function continue to inspire further scrutiny toward optimizing well-being (Grahl et al. 2018).

Additionally, the methods of processing and extraction have a big impact on the concentration of these compounds. The alcoholic and aqueous extracts of *S. platensis* include 205 mg and 334 mg of gallic acid, respectively, per 100 g dry weight. Yields depend on the choice of solvent and based on a comparative study acetone extracts have the highest phenolic yields (7.37 mg/g), followed by methanol (3.46 mg/g) and ethanol (2.28 mg/g) (Matos et al. 2020; Salamatullah et al. 2014). The highest amount phenolic compound was pyrogallol; with the highest amount at 638.50 mg/100 g in *Spirulina* biomass and 12.33 mg/100 g in aqueous extracts respectively. Hesperidin is the most abundant flavonoid in biomass (9.013 mg/100 g), followed by ethanol (0.652 mg/100 g) and aqueous (0.359 mg/100 g) extracts (Gabr et al. 2020).

Another environmental factor, pH can affect compound production. When the cultivation conditions were optimized (pH 10), flavonoids contents in ethanol extracts (7.6 mg/g) and phenolic contents in acetone extracts (0.52 mg/g) significantly increased (Chaiklahan et al. 2012). Moreover, food products fortified with *Spirulina* have been found to exhibit greater antioxidant activity. Generally, phenols present in food, for example, 1.6–1.7 mg gallic acid/g of spirulina have been reported for biscuits containing 6% *Spirulina*, showing its potential for food preservation (Batista et al. 2019).

### Tannins, saponins and quinones

Tannins, particularly ellagitannins, are a phenolic compound renowned for their plethora of health advantages. They exhibit potent antioxidant and anti-inflammatory abilities, helping to diminish the prospects of cardiovascular diseases, certain cancers, and other ailments. Gallo-tannins display analogous virtues and can inhibit the proliferation of colorectal cancer while also treating symptoms such as digestive issues (El Nakib et al. 2019). Saponins are glycoside compounds with surfactant properties that improve nutrient absorption and help emulsify fats. The total saponin content of saponins in *Spirulina* is 28 mg/g dry weight (Akbarizare 2019). A notable saponin, spirulan, modulates immune response. They have antipathogenic effects by disrupting the cell membranes of pathogens, which inhibits their growth. These are the detergents, which can decrease the voltage between the bacterial cell wall and permeabilize the membrane (Bortolini 2022). Additionally, saponins can bind to bile acids, promoting their excretion and assisting in cholesterol management, potentially leading to lower blood cholesterol levels. Furthermore, saponins are known to enhance immune function by boosting the activity of immune cells, thereby strengthening the body defenses against infections (Sharma et al. 2021). Quinone compounds can bind to cell proteins, rendering them dysfunctional, thus disrupting cell metabolism (Ilieva et al. 2024).

### Terpenoids

*Spirulina* sp. is loaded with different types of terpenoids like monoterpenes, sesquiterpenes and triterpenes, all providing anti-inflammatory, antimicrobial, antioxidant (Siddiqui et al. 2024). Key terpenoids, for example, alpha-amyrin and beta-amyrin, both pentacyclic triterpenes, are recognized for their anti-inflammatory properties. Squalene is another significant component that promotes skin wellness and has antioxidant effects (Huang et al. 2009). Moreover, limonene, a monoterpene, displays promise for anti-cancer and anti-inflammatory advantages (Kathem et al. 2024). These terpenoids can damage the porins in the outer membranes of bacterial cell walls through the formation of a robust bonding polymer, thus hindering the flow of nutrients and giving antibacterial properties (Ilieva et al. 2024).

### Pharmacological properties of *Spirulina* sp.

#### Antibacterial activities

**Fig. 2:**
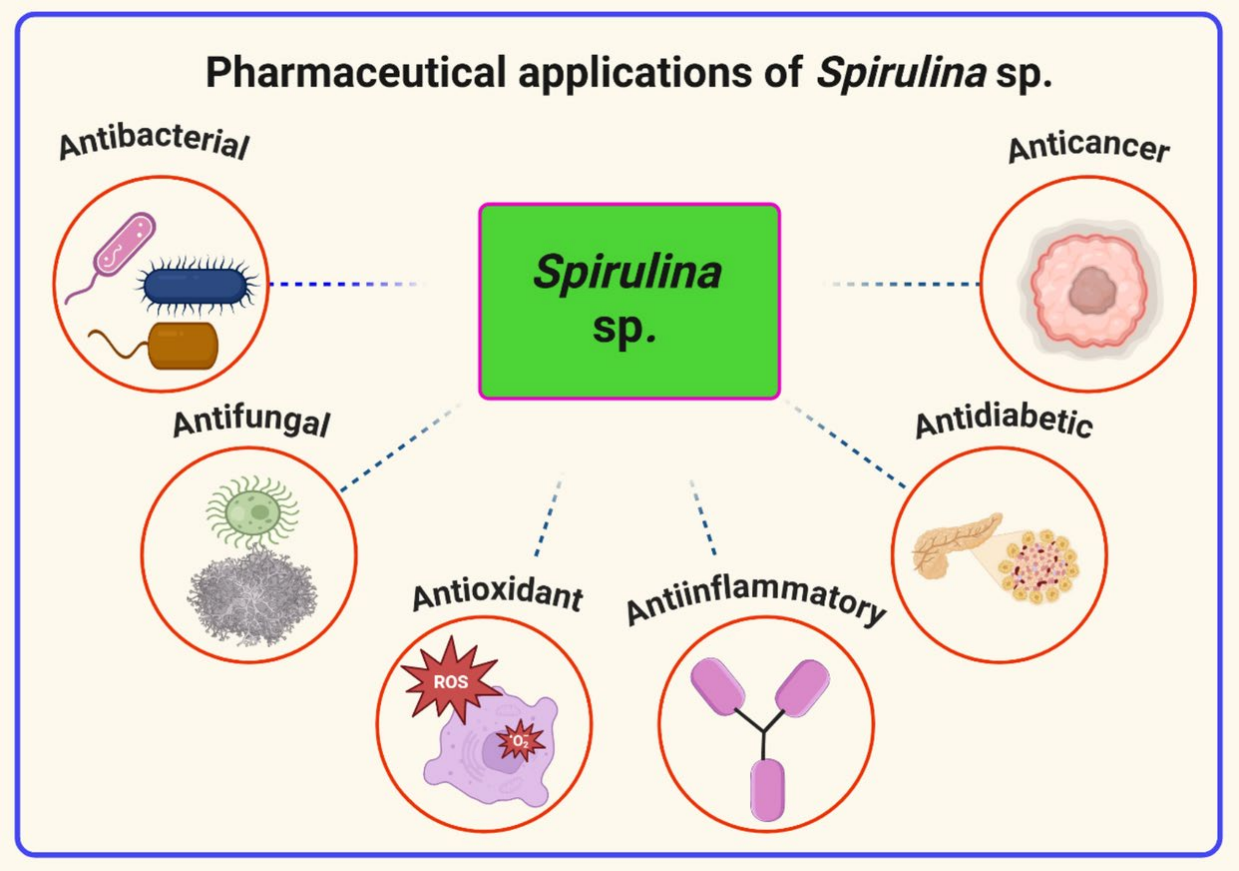
*Spirulina* sp. as a pharmaceutic compound

The incidence of bacterial resistance rising steadily is making some diseases incurable. *Spirulina* sp. extracts and microbial metabolites have promising in exhibiting potent antibacterial, antifungal, antiviral, and anticancer activity against resistant strains mentioned in (Table 3, Fig. 2). Mutants, methicillin-resistant Staphylococcus aureus and vancomycin-resistant *Enterococci* have been commonly known worldwide nowadays. The screening of cyanobacteria proves to be a promising source for drug–resistant bacteria, including both Gram-positive and negative, by interrupting bacterial protein synthesis or slowing down their metabolism, affecting bacterial growth. (Mandhata et al. 2023).

**Table 3:** Antibacterial, antifungal, and anticancer properties of *Spirulina* sp.

Organism	Active compound/extract	Bacterial/fungal/viral targets and type of cancer	Mode of action	References
*S. maxima*	Lipophilic and phenolic compounds	Antibacterial activity against *S. aureus*, *E. coli* and *B. cereus*	Disrupts bacterial cell membrane,	Pina-Pérez et al. (2017)
	C-Phycocyanin	Antifungal activity of Candidiasis	Inhibits fungal growth	Dananjaya et al. (2020)
	Selenium	Antifungal activity against of Aspergillosis	Disrupts the fungal cell wall	Saied et al. (2023)
	Purified extract mainly containing Phenols	*P. oxalicum, F. solani, R. solani*	Inhibits fungal growth	Battah et al. (2014)
	Hot water extract	HSV-2, other enveloped viruses	Blocks viral infectious cycle at absorption and penetration	Liang et al. (2021)
	L-AsnA	Antiviral activity against of CSB3 virus	Inhibits viral replication cycle	
*S. fusiformis*	Phycobiliproteins	Antibacterial activity against of *S. pyogenes*, *S. aureus*, *S. typhi, P. vulgaris, E. coli, S. marcescens*	Inhibits bacterial metabolism	Hamad et al. (2023)
	Antioxidants	Antifungal activity against of Candidiasis, *A. niger* and *a. flavus*	Reduces oxidative damage in infected cells	
	Bioactive extract	Tinea (ringworm)	Enhances immune response	Ilag. (2018)
*S. platensis*	Methanolic extract	Antibacterial activity against of *S. aureus*, *P. aeruginosa*, *B. cereus*, *L. monocytogenes*, *E. coli*, *S. typhi*, *K. pneumoniae*	Disrupts bacterial growth	Abdel-Moneim et al. (2022)
		Antibacterial activity against of *V. parahaemolyticus*, *V. alginolyticus*	Inhibits bacterial growth	Abdulmumin A. Nuhu et al. (2013)
	*Spirulina* ethanolic extract	Antibacterial activity against of *S. aureus* and *P. aeruginosa*	Inhibits bacterial growth	Hardiningtyas et al. (2022)
	Phycocyanin	Antifungal activity of Candidiasis	Disrupts fungal cell wall synthesis	Righini et al. (2019)
	β-Carotene	Antifungal activity against of *C. albicans*	Antioxidant properties reduce oxidative stress	El-Sheekh et al. (2010)
	Polysaccharides	Antifungal activity of Candidiasis	Enhances immune response	Marangoni et al. (2017)
	Polysaccharides	Antifungal activity of aspergillosis	Inhibits fungal growth	Al-ghanayem et al. (2017)
	Calcium spirulan	Antiviral activity against of HCMV, HSV-1, measles virus, mumps virus, HIV-1, influenza virus	Inhibits replication of enveloped viruses	Lee et al. (2001)
	Allophycocyanin	Antiviral activity against of Enterovirus 71	Delays viral RNA synthesis	Shih et al. (2003)
	Aqueous extract	Antiviral activity against of HIV-1, HSV-1	Inhibits viral replication in PBMCs and human T-cell lines	Khan et al. (2005a, b)
	C-phycocyanin	Antiviral activity against of SARS-CoV-2	Inhibits NSP12, interfering with virus replication	Raj et al. (2020)
	Phycocyanin	Anticancer activity of Liver	Induces apoptosis in liver cells	Ramkrisnan et al. (2013)
	*Spirulina* Phycocyanin	Oral—squamous cell carcinoma	Inhibits cell proliferation	
	*Spirulina* Dunaliella Extract	Squamous cell carcinoma (DMBA induced)	Promotes immune response	
	*Spirulina* Dunaliella Extract (oral)	Oral buccal pouches tumor	Enhances cytokine production	
	*Spirulina* Polysaccharide extract	Hepatoma	Modulates immune signaling	
	Water extract	HeLa cells	Induces cell cycle arrest	
	Whole *Spirulina* feed	Colon	Reduces oxidative stress	
	*Spirulina* supplementation	Oral leukoplakia	Scavenges free radicals	
	Ca- Spirulan	Lung cancer	Inhibits metastasis	
	*Spirulina* C-Phycocyanin	Leukemia	Triggers apoptosis	
	Phycocyanin	Human melanoma	Inhibits cell growth pathways	
	Hot water extract	B16 melanoma	Activates natural killer cells	
	*S. Maxima* ultrasonic extraction	Lung, liver, stomach, and breast cell lines	Modulates gene expression	
	*S. platensis* extract	Squamous cell carcinoma	Induces apoptosis in tumor cells	
	Polysaccharides from S. platensis	B16 melanoma cells	Stimulates macrophage activation	
	β-Carotene	Skin cancer	Reduces inflammation	
	Curcumin	Prostate cancer	Inhibits NF-κB signaling	
	Resveratrol	Breast cancer	Activates apoptotic pathways	
	Quercetin	Ovarian cancer	Disrupts cancer cell migration	
	Green tea extract	Pancreatic cancer	Modulates cell cycle progression	

NB: *Staphylococcus aureus, S. aureus*; *Escherichia coli, E. coli*; *Bacillus cereus, B. cereus; Spirulina maxima*, *S. maxima; Pencillium oxalicum, P. oxalicum; Fusarium solani, F. solani; Rhizoctonia solani, R. solani;* Pseudorabies virus, PRV; Human cytomegalovirus, HCMV; *Streptococcus pyogenes, S. pyogenes*; *Salmonella typhi, S. typhi*; *Proteus vulgaris, P. vulgaris*; *Serratia marcescens, S. marcescens*; *Aspergillus niger*, *A. niger*; *Aspergillus flavus*, *A. flavus*; *Pseudomonas aeruginosa*, *P. aeruginosa*; *Listeria monocytogenes*, *L. monocytogenes; Klebsiella pneumoniae, K. pneumoniae; Vibrio parahaemolyticus*, *V. parahaemolyticus; Vibrio alginolyticus, V. alginolyticus; Spirulina fusiformis, S. fusiformis; Spirulina platensis, S. platensis*

*S. maxima* extracts had been reported as a potential alternative to combat the drug-resistant bacteria due to its high antibacterial effectiveness. The maximum antibacterial potency was given by the extract of *S. maxima* with higher MIC of 30 µg/ml. The antimicrobial effectiveness of it is probably related to the occurrence of lipophilic and phenolic compounds, which can damage bacterial cell membranes, disrupt bacterial metabolism and provide antioxidant protection that further contributes to the overall antibacterial effect (Pina-Pérez et al. 2017).

Other *Spirulina* species with good antibacterial activity in addition to *S. maxima* include *S. fusiformis* and *S. platensis*. These phycobilliproteins were more potent against gram negative bacteria such as *Salmonella typhi, Serratia marcescens* and *Proteus vulgaris,* when extracted from *S. fusiformis*. On the other hand, the methanolic extract of *S. platensis* was active against both Gram-positive and Gram-negative bacteria, including *Bacillus cereus, Staphylococcus aureus, Listeria monocytogenes, E. coli, Salmonella typhi,* and *Klebsiella pneumoniae*, showing inhibition zones from 17 to 22 mm at 10 mg/mL. Noteworthy, its ethanolic extract displayed greater activity against *Staphylococcus aureus* and *Pseudomonas aeruginosa* associated with chitosan nanoparticles than with pure extracts. Other findings showed that *S. plantensis* had exceptionally potent antibacterial activity against *Vibrio parahaemolyticus, Vibrio anguillarum, Vibrio splendidus, Vibrio scophthalmi, Vibrio alginolyticus,* and *Vibrio lentus*. The bioactive compounds identified were fatty acids, pigments, and peptides which disrupt cellular functions and inhibit bacterial growth (Nuhu 2013).

Antibacterial activity of *Spirulina* acetone extracts also demonstrated noticeably excellent antibacterial activity against *Klebsiella pneumoniae* (41 mm inhibition zone) further emphasizing the antibacterial potential of *Spirulina* sp.. In fact, this extract was more effective than the common antibiotic kanamycin, the presumed mechanism being disruption of microbial membranes by phenolic compounds. Polysaccharides present in *Spirulina* extracts have also been shown to have hyaluronidase inhibitory activity and immunomodulatory activity. Its broad-spectrum antibacterial property on gram-positive cocci and gram negative bacilli are thought to be due to the presence of fatty acids and polar solvent soluble lipid molecules. On the other hand, only *Staphylococcus aureus* was slightly sensitive to aqueous extract of *Spirulina* with an inhibition zone of 8.5 mm while the tested strains were resistant to this extract. The significant gap in potency between acetone extracts and aqueous extracts may indicate that many phytochemicals are more soluble in organic solvents such as acetone and diethyl either than in aqueous or methanolic solvents (Nabti et al. 2023).

#### Antifungal activities

*Spirulina* sp. can be considered as a promising source of antifungal agent that is seen to reduce fungal diseases. Alkaloids are known for their diverse biological activities, including antifungal properties, and their presence often correlates with effective antifungal action (Ilieva et al. 2024). The presence of various peptides, phenolic compounds and secondary metabolites in *S. maxima* extract are seen to reduce the fungal growth against *Fusarium solani*, *Rizoctonia solani*, *Candida albicams, Aspergillus niger* and is seen to be most potent against *Penicillium oxalicum* (Battah et al. 2014). *S*. *platensis* methanolic extract inhibited glucosamine production up to 56% in *A*. *niger.* Activity against *C. albicans* can be seen both in *S. fusiformis* and *S. platensis* extract (Nuhu 2013). *E*xperimentally it was concluded that acetone extract showed maximum antifungal activity against *Fusarium culmorum* with 34 mm inhibition diameter (Nabti et al. 2023).

#### Antiviral activities

Analysis of potential bioactive compounds of *Spirulina*, have elucidated mechanisms by which the viral life cycle may be inhibited at several stages (Table 1). Methanol–water (3:1) extracts of *S. maxima* can inhibit the HSV-2 in its absorption and penetration phase thus interrupting the virus cycle. L-AsnA is also found to possess potential antiviral activity against CSB3 virus. *Spirulina* hot water extract shows a similar inhibitory effect on several stages of the viral life cycle, on Vero cells by blocking the absorption and penetration stages. This presumably restricts the ability of the virus to attach and enter host cells by molecular means such as masking viral receptors or changing cell surfaces properties making virus entry more difficult (Liang et al. 2021).

Calcium spirulan (Ca-SP) found in *S. platensis*, due to sulphated polysaccharides component, has a strong potential as an antiviral agent. It prevents replication of several enveloped viruses, including HCMV, HSV-1, measles, mumps, HIV-1, and influenza through inhibition of viral entry, fusion, or replication. The disruptive mechanism of ethanol on viral structure notably inhibits the entry of many non-enveloped viruses, often the more environmentally robust viruses compared to the enveloped viruses including astrovirus type 1, Coxsackievirus, adenovirus type 7, rotavirus Wa strain, and adenovirus type 40. This mechanism involves disrupting the viral capsid or the interaction between ethanol and viral surface proteins. Though these viruses possess no lipid envelope, ethanol can still damage them. Moreover, the *Spirulina*-derived phycobiliprotein allophycocyanin has exhibited potentials of a non-toxic and affordable antiviral agent through blocking RNA synthesis in some viruses including Enterovirus 71, a virus responsible for the life-threatening hand, foot and mouth disease of children (Liang et al. 2021). Moreover, *Spirulina* had shown antiviral activity against SARS-CoV-2 by blocking the active site of NSP12 (non-structural protein 12), an essential component for viral RNA amplification. Bioactive compounds such as C-phycocyanin and allophycocyanin may bind with the NSP12 and hinder the binding, entrance and replication of the virus. In addition, *Spirulina* has antioxidant properties, which also enhance the immune response of the host. Furthermore, extracts of *S. fusiformis* in phosphate buffer and hot water also exhibited anti-herpetic activity against HSV (Sharaf et al 2010).

#### Antioxidant activities

The high antioxidants present in *Spirulina*, attributed due to phycocyanin and allophycocyanin is essential for scavenging free OH radicals and protecting live cells against oxidative stress (Campos et al. 2024). The hepatoprotective properties observed in *S. maxima* are primarily attributed to its antioxidant activity, which is seen to lessen the liver damage and promote liver health (Pérez-Juárez 2022). Moreover, it is effective against a series of potentially toxic compounds such as an insecticide deltamethrin, and antibiotics tilmicosin, gentamicin, and erythromycin. *Spirulina* has shown potential to enhance antioxidant defenses and modulate the key transcription factors. The antioxidant action is achieved through the activation of Nrf2, which leads to an increased production of the body's primary antioxidant glutathione and also promotes the transcription of key antioxidant enzymes like SOD, CAT, GPx. Moreover, it hinders the ARE and NF-κB signaling pathways, leading to lower generation of pro-inflammatory factors. Through effects on neurochemicals, *Spirulina* results in increased brain and serum manganese levels that have vital roles either as enzymatic co-factors or synthesize neurotransmitters. It also elevates MDA, a biomarker of lipid peroxidation, and decreases acetylcholine, GPx, and dopamine levels (Ibrahim et al. 2015). The carotenoids, omega-3 fatty acids, C-phycocyanin and allophycocyanin contents of *S. maxima* were suggested to have protective actions against Simvastatin-induced hyperlipidemia via reduced total liver lipids, lower levels of liver triacylglycerols, and diminished serum triacylglycerols. It also has the capacity to increase antioxidant defenses, reduce inflammation and modulate neurotransmitter systems as well as provide neuroprotective support. Additionally, supplementing it has demonstrated potential in reducing the ROS damage that cause to the brain in neurodegenerative diseases like Alzheimer's disease, Parkinson's disease, Huntington's disease, Amyotrophic lateral sclerosis (ALS) and heart stroke (Sinha et al. 2020).

#### Nephroprotective activities

*S. fusiformis*, through its radical scavenging properties, gives kidney protection against injury induced by gentamicin exposure. It does this by countering the ROS mitochondrial damage, counteracting inflammation/programmed cell death as well as alleviating oxidative and nitrosative damage. Previously administered *Spirulina* sp*.*, even without interfering with the metabolism of cyclosporine CsA can reduce both renal dysfunction and structural changes associated with cyclosporine-induced renal lesions by increasing levels MDA in kidneys while lowering lysosome enzyme activity in renal tissues (Khan et al. 2005a, b). Furthermore, *Spirulina* has been shown to prevent nephrotoxicity induced by both cyclophosphamide and cisplatin. This is due in large part to its ability to easily neutralise hydroxyl and peroxyl radicals as well as peroxynitrite and superoxide radicals, with the help of a phytochemical called phycocyanin (Sinanuglu et al. 2012).

#### Cardioprotective activity

*Spirulina* sp. helps to reduce lipid peroxidation, decrease the mortality rate, normalizes the antioxidant enzymes like SOD, CAT, GPx and decreases problems due to free radical damage, mutagenesis, alteration in mitochondrial function and caspase-mediated cell death caused due to doxorubicin-induced cardiotoxicity (Nuhu 2013). Research suggests that it can reduce hypertension, improve cholesterol profile and promote cardiac well-being.

The phycocyanin present in *Spirulina* cells is a key component to combat oxidative stress and inflammation, which are significant contributors to cardioprotective diseases. It is seen to reduce oxidative damage and protect the endothelial cells that line the blood vessels, promoting better circulation and heart function. Additionally, it enhances the lipid profiles by decreasing the LDL and triglycerols and increasing the HDL, minimizing the possibility of atherosclerosis and other heart-related issues (Pandey and Singh. 2022). Moreover, studies indicate that *Spirulina* sp. has anti-inflammatory effects that promote cardiovascular health by reducing chronic inflammation that can lead to heart disease (Shiri et al. 2024). *Spirulina* sp. has pointed out its advantages in respect to BP, lowering systolic pressure and stiffness index leading to lower blood pressure and reduced arterial stiffness which lowers heart disease risk (Carrizzo et al. 2020).

*Spirulina* could be interesting as an add on therapy for patients who are not well controlled on BP medications such as ACE inhibitors. Two tripeptides (Ile-Gln-Pro and Val-Glu-Pro) with antihypertensive activity have been recognized *from S. platensis* and result in a significant decrease in systolic and diastolic BP. The peptides also diminish indices of ventricular mass, which is especially advantageous with the treatment of ventricular hypertrophy. These tripeptides have a strong impact on the RAS, an important regulator of body fluid volume and blood pressure. They induce alterations in RAS expression in heart tissues, decreasing angiotensin II, the most powerful vasoconstrictor, in the tissues, responsible for hypertension and cardiac hypertrophy. Such action ultimately enhances heart function and protects it against structural complications that often appear in chronic hypertension, such as hypertrophy and cardiomyopathy (Pan et al. 2015). A 3 month clinical trial including 30 patients with ischaemic heartdisease and hypercholesterolaemia showed a 22.4–33.5% reduction in total cholesterol, of triglycerides reduction by 6.4 mg and LDL-c by 7.1 mg. (DiNicolantonio et al. 2020).

Additionally, Silicon-supplemented *Spirulina* sp. regulates collagen production for greater arterial wall elasticity, improving vascular compliance by enabling blood vessels to contract and relax more easily. *Spirulina* sp. supplementation has also been documented to decrease the endothelial damage biomarkers sVCAM-1, sE-selectin, and endothelin-1, and increase glutathione levels and glutathione peroxidase activity, improving vascular angiogenesis and attenuating hypertensive effects (Prete et al. 2024).Furthermore, *Spirulina* aqueous extracts are known to include twelve peptides which enhance aortic vascular activity leading to attenuated superoxide generation, enhanced heme oxygenase-1 and phosphorylated eNOS levels, and nitric oxide release (Prete et al. 2024).

#### Anticancer activities

*Spirulina* sp. is one of the promising natural sources showing anti-tumour activities because of the presence of phenolic and alkaloids in it. Such compounds have remarkable potential to disrupt cell cycle progression, induction of apoptosis and critical signaling pathways involved in cancer cell survival. Conventional cancer chemotherapeutic drugs mainly cause severe adverse conditions including alopecia, diarrhea, mouth sores, queasiness, puking, and loss of appetite. The *Spirulina* extracts may alleviate most of these adverse effects, which could make it a supplement to treatment. Crude extract of *S. platensis* had been observed to inhibit the growth of human mammary adenocarcinoma clonal cell population (Fayyad et al. 2019). Studies documented that the methanolic extract of *S. platensis* has anti-tumour activities against different human neoplastic cell models, such as its efficacy against Ehrlich Ascites Carcinoma (EAC). Hepatocellular carcinoma (HCC) indeed tops the list as the most common kind of liver cancer and also comes as the sixth-most common neoplasm worldwide. Contributing factors include infections with HBV and HCV viruses, alcoholic liver disease, and NAFLD. Chronic infection with either HBV or HCV viruses accounts for most of the cases of HCC throughout the world. Obesity and insulin resistance are newer/emerging risk factors for HCC due to their association with NAFLD (Akbarizare et al. 2020).

Its aqueous and methanolic extract yield promising results against HepG2 liver cancer cells in a concentration-dependent manner. Studies suggested that the bioactive compounds, mainly alkaloid over phenolic, exhibited a higher cytotoxic effect than crude extract against HepG2 liver cancer cells, with a three times greater potency. Furtermore, studies also document the effect of extracts and some bioactive compounds of *S. platensis* in other cancer cell lines like Kasumi-1, K562, pancreatic cancer cells, HCT116 colon carcinoma cells, and McF-7 breast cancer cells (Fayyad et al. 2019).

The chemopreventive properties of *S. fusiformis* were seen to hinder the development of oral malignancy and conditions like leukoplakia among pan-tobacco chewers. Also, the experimental survey concluded that there was a regression of oral leukoplakia lesions in individuals consuming *Spirulina* (Grawish et al. 2010). It is also found effective in colon carcinogenesis. Studies showed that it effectively decreased the formation of abnormal crypts in the colon of rats, which are precursors to colorectal cancer, even though when treated with, DMBA, the chemical inducing colon cancer. Additionally, intravenous injection of the photosensitizer, radachlorin, obtained from *S. plantensis* induced complete regression of tumors. Phycocyanin compound is seen to show anticancer activity against liver cancer and cytostatic activity against oral-squamous cell carcinoma. *Spirulina Dunaliella* extract does total tumour regression in DMBA-induced squamous cell carcinoma and also causes an immune response against oral tumours (Grawish et al. 2010). *Spirulina* Polysaccharide extract inhibited the explosion of artistic hepatoma cells against hepatoma cancer. Water extract shows cytotoxic activity against HeLa cells and whole *Spirulina* feed is seen to be effective against colon cancer. Ca-SPs are effective against lung cancer and show anti-heparanase activity, inhibiting metastasis and invasion. C-PC is effective against caspase-dependent leukemia. Se- PC is seen to be effective against A375 melanoma cell line and MCF-7 mammary adenocarcinoma cells. Hot water extract is effective against B16 melanoma showing natural killer cells dependent tumoricidal activity (Ramakrishnan 2013).

Heteropolysaccharides from *Spirulina* are showing great promise in cancer research and boosting the immune system. Research suggests that these compounds can significantly slow the expansion of A549 lung tumor cells, indicating a potential anti-cancer effect. Moreover, these heteropolysaccharides enhance immune function, especially in macrophages. They encourage both the growth and phagocytic ability of these immune cells, which are vital for the body's defence against infections and cancer. Additionally, they promote the release of key cytokines, such as nitric oxide, interleukin-1 beta, and TNF-α, all of which are important in inflammation and immune responses (Cai et al. 2022).

#### Neuroprotective activities

Due to inherent antioxidant properties, *Spirulina* sp. is said to neutralize oxidative stress and thus offer protection against the neurobehavioral damaging results of systemic kainic acid with a possible decrease in KA-neuronal mortality in the CA3 hippocampal region (Pérez-Juárez et al. 2016). Additionally, it was reported to be helpful in Parkinson's disease by inhibiting the rotational behaviour mediated by apomorphine. This inhibition suggests that *Spirulina* may be useful in managing the motor dysfunction caused because of the disease. Its treatment protect against the depletion of dopamine and DOPAC, both being crucial for normal motor activity. The treatment also decreased the levels of nitrite and TBARS, which are markers for oxidative stress and lipid peroxidation, respectively. Additionally, the immunoreactives for iNOS and COX-2 were also downregulated following *Spirulina* treatment. (Bermejo-Bescós et al. 2008). Inductive iron has been shown to reduce the toxicity caused in the SH-SY5Y neuroblastoma cells by *S. platensis* protein extract (Lopes et al. 2022). The neuroprotection exerted by the protein-rich portion from *S. platensis* in hemiparkinsonian rats arises through the downregulation of brain inflammatory-related enzymes and glial fibrillary acidic protein It protect against aluminium chloride-induced neural degeneration. (Yousef et al. 2020).

The neuroprotective effects of *S. platensis* are enhanced following a high-pressure homogenization process (Choi et al. 2018a, b). *S.fusiformis* showed parkinsonism effect in 6-OHDA induced rat model (Chattopadhyaya et al. 2015)*.* This includes improvements in antioxidant enzyme activities, elevated levels of tissue TNF-α, IL-17, and NF-κB, as well as inhibition of Tau protein through phosphorylation (Trotta et al. 2022). *S. platensis* has been shown to exert neuroprotective efficiency against the changes in plasma neurochemistry, of catecholamines like adrenaline, noradrenaline, dopamine, serotonin, gamma-aminobutyric acid and acetyl cholinesterase activity in rats induced by cadmium. Cadmium triggers oxidative stress and disrupts the brain cell membrane undergoing DNA fragmentation which is the pathological feature of brain tissue damage. Therefore, the findings conclude that *S. platensis* probably has an important role in alleviating brain injury and protecting against cadmium chloride toxicity (Alam and Hendawi. 2015). The ultrasonic extraction with antioxidant activities of the fermented *S. maxima* improved the neuroprotective effect (Choi et al. 2018a, b). It has been documented that *S. maxima* 70% ethanolic extract alleviates the deficit in memory and learning (Koh et al. 2017).

#### Antiaging activities

Studies done on phycocyanin proved that, a drug prepared from *Spirulina* extract, helps in oxidative stress reduction in mammals and the yeast, *Yarrowia lipolytica*. Experimentally, cells treated at inoculation with phycocyanin survived longer than control and also had higher levels of ROS. This ironic observation points out that perhaps the use of phycocyanin could stimulate the survival mechanisms by a slight increase in oxidative stress (Nova et al. 2024). The methanolic extract of *S. platensis* boosted telomerase activity by 139%, while exhibiting significant toxicity to MCF-7 cells (82%), with an IC50 of 0.035 mg/ml retarding the ageing process. The aqueous extract also presented anti-ageing activity on HDF cells by enhancing telomerase activity at 0.004 mg/ml by 84%. All the extracts of *S. platensis* except the aqueous extract are presented to inhibit MCF-7 cancer cells through a mechanism which is not identical to telomerase inhibition (Akbarizare 2021). Other than this *Spirulina* sp. has various application (Table 4).

**Table 4:** Active agents of Spirulina sp. and their application in preventing various diseases

*Spirulina* sp.	Active compounds	Sample type, dosage, organism	Potential applications	Diseases that can be prevented	References
*Spirulina* inhibits zymosan-induced arthritis	C-phycocyanin	Alcoholic extract; 50 mg/kg; Rats	Development of arthritis concealed; Helps in microscopic and histopathological lesions in rats	Rheumatoid arthritis	Ali et al. (2015)
Ameliorates psychosis-like symptoms induced by dizocilpine	Phycocyanin extract	Aqueous extract; 180 mg/kg; Rats	Helps in hepatorenal toxicity and cognitive behavior	Psychosis-like symptoms	Haider et al. (2021)
Delay or inhibit the occurrence and development of atherosclerosis.Improves endothelial function and reduces stiffness	C-phycocyanin	1 g per kg bodyweight (BW) per day of freeze-dried S. platensis; mice and also fresh spirulina extracts in humans	C-PC promotes the expression of CD59 gene	Atherosclerosis	Strasky et al. (2013)
Reduces inflammation and modulates immune response	Phycocyanin, antioxidants	Fresh weight; 300 mg/kg; Rodents	Treatment of inflammatory conditions	Inflammatory bowel disease	Liu et al. (2016)
Protects liver cells and promotes detoxification	C- Phycocyanin, Phycocyanin and essential fatty acids	Fresh extract;400 mg/kg of PC; Mice	C-PC promotes the proliferation of serum T cells, inhibits the levels of serum alanine amminotransferase, aspartate amminotransferase, malondialdehyde, etc., and increase the expression of superoxide dismutase in the livers	Hepatitis, fatty liver disease,ethanol induced acute liver injury and alcoholic liver diseases	Ansari et al. (2023)
Enhances lung functioning	Phycocyanin(PC)	Dry weight; 50 mg/kg; Mice	PC inhibits the level of inflammatory factors in lung tissue, increases the relative expression of antioxidant enzymes, and down-regulates the activation of TLR2/MyD88/NF-κB pathway	Pulmonary fibrosis	Liu et al. (2016)
Improves insulin sensitivity and blood sugar regulation	Phycocyanin, phenolic compounds	Clinical trials ranging from 2 g/day to 8 g/day, in the form of capsules, powder, or tablets, (45 days to 3 months); Animal studies, with dosages from 5 mg/kg to 50 mg/kg biomass or purified extracts (e.g., phycocyanin, phycocyanobilin, phycopeptides)	Both Human trials and Animal studies showed significant improvements in fasting blood sugar and improved insulin sensitivity	Diabetes mellitus type 2	Prete et al. (2024)
Protects neurons from oxidative damage	Phycocyanin, antioxidants	70% ethanol extract form; male ICR mice with scopolamine-induced memory deficits, and, Sprague–Dawley adult male rats with cerebral ischemia; 50–200 mg/kg dosage	It support brain health, protect against ischemic injury, and improve cognitive function	Alzheimer’s, Parkinson’s disease	Trotta et al. (2022)
Promotes tissue repair and positive effects on innate immunity	Polysaccharides and phycocyanin	Water-soluble extracts; dosages range from 50 to 200 mg/kg in mice and chickens	Polysaccharides and phycocyanin enhance immunity by stimulating erythropoietin levels, improving tumoricidal activity of NK cells, stimulating interleukin secretion from macrophages, strengthening the immune response, promoting bone marrow reproduction, thymus growth and spleen development,	Various immune disorders	Khan et al. (2005a, b)
Lowers blood pressure through vasodilation	Peptides, phycocyanin	Supplied orally; 4.5 g/day, (6 weeks); Humans	Treatment of hypertension	Hypertension	Torres-Duran et al. (2007)
Modulates allergic reactions and reduces histamine	Phycocyanin	Dry weight; 2000 mg/day (2 months); Humans	Treatment of allergies and asthma	Allergic rhinitis	Batista et al. (2021)
Improves lipid and glucose metabolism	Antioxidants	Powdered form; 2 g/day; Human trials	Management of metabolic syndrome	Metabolic syndrome, obesity	DiNicolantonio et al. (2020)
Moisturizes skin and promotes healing	Essential fatty acids	Fresh extract and Hot water extracts; Topical application; Human	Treatment of dry skin, dermatitis	Eczema, psoriasis	Ragusa et al. (2021)
Removes heavy metals (Renal toxicity induced by mercury) and toxins	Phycocyanin	Powder and water extract; 10 g/d; Rats	Detox programs and liver health	Heavy metal toxicity	Fukino et al. (1990)
Supports memory and cognitive performance	Antioxidants, Memory dysfunctions, amyloid-β deposition, oxidative stress biomarkers	Alcoholic extract; 50, 200 mg/kg/day, for 12 weeks; Rodents	Cognitive health and memory enhancement	Cognitive decline, dementia	Sorrenti et al. (2021)
Aids in weight management	Phycocyanin, dietary fiber	Dry weight; 1–2 g/day; Human trials	Weight loss and obesity management	Obesity, metabolic syndrome	Alfadhy et al. (2022)
Enhances lung function and alleviates asthma	Phycocyanin, polysaccharides	Alcoholic extract; 1 g/day; Human trial	Treatment of respiratory conditions	Asthma, COPD	Labhe et al. (2001)
Promotes gut bacteria and aids digestion	Dietary fiber, oligosaccharides	Dry weight oral composition; 1.5 g/kg-3.0 g/kg; Human trials	Treatment of gastrointestinal disorders	IBS, dysbiosis	Hu et al. (2019)
Protects retina and improves vision	Carotenoids, zeaxanthin	*Spirulina*-supplemented solid diet, which contained 20% of it; 0.6 g/d; Mice	Prevention of age-related macular degeneration	Macular degeneration, retinal disorders	Okamoto et al. (2019)
Exhibits antiviral activity	Polysaccharides	Hot water extract; 2 mg/ml; Human clinical trials	Support in viral infection management	Viral infections (e.g., herpes simplex virus 2,)	Hernández-Corona et al. (2002)
Supports reproductive health and fertility	Essential fatty acids, antioxidants	Fresh weight; with 10–30% (w/w) addition in diets; Rats	Treatment of infertility	Infertility	Salazar et al. (1996)
Improves bioavailability of nutrients	Polysaccharides	4% w/w addition to food; Rats	Supports overall nutrition and health	Nutrient deficiencie, malnutritin in children	Kumar et al. (2023)
Protects kidney function	Antioxidants, amino acids	Aqueous extract; 1000 mg/kg bw/day; Mice	Management of kidney diseases	Chronic kidney disease	Sinanoglu et al. (2012)
Balances lipid levels	Phycocyanin, fiber	Powdered form; 2–4 g/day; Human trials	Cardiovascular health and metabolic management	Dyslipidemia, cardiovascular diseases	Deng and Chow (2010)
Enhances mood and alleviates depression	Omega-3 fatty acids, tryptophan	Powder form; 400 mg/kg; Rats	Treatment of depression and anxiety disorders	Depression, anxiety disorders	Basavarajappa et al. (2023)

#### Anti-inflammatory activities

Research suggests C-PC's impact on inflammation and oxidative damage underscoring its potential as both a therapeutic and anti-inflammatory agent. Those treated with C-PC saw a notable decrease in serum levels of TNF-α, a crucial proinflammatory cytokine involved in various inflammatory diseases. Additionally, C-PC exhibited inhibitory effects on COX-2, an enzyme frequently overexpressed during inflammation and known to play a role in producing pro-inflammatory intermediates (Reddy et al. 2003). The treatment also caused increased levels of oxidative stress markers in the liver, kidney, and brain tissues, including MDA, NO, SOD, catalase, glutathione, and GPx, revealing C-PC may aid in reducing inflammation, and affects oxidative stress pathways. The commencement of the Nrf2 pathway in pancreatic beta cells suggests that C-PC encourages antioxidant responses, as Nrf2 regulates antioxidant protein expression. Simultaneously, the inhibition of the NF-κB pathway points to a decrease in inflammatory signaling, contributing to the observed decrease in proinflammatory cytokines (Liu et al. 2022).

Evidence suggests that *Spirulina* sp. displays anti-inflammatory properties alike diclofenac sodium, a recognized anti-inflammatory medication, which implies *Spirulina* could function as an effective natural substitute for addressing inflammation (Nipa et al. 2020). Moreover, a current meta-analysis of managed clinical experiments uncovered that *Spirulina* dietary supplementation meaningfully lowered interleukin-6 levels in people with a BMI underneath 25 kg m^−2^. Since interleukin-6 is often found at elevated levels in diverse inflammatory conditions, decreasing its concentration can advantage overall health. Recent studies suggest *Spirulina* dietary supplementation may reduce inflammation and muscle harm brought about by exercise. Specifically, it is seen to diminish levels of creatine kinase, C-reactive protein, and F2-isoprostane both quickly and 24 h post-exercise amongst elite rugby players. Thus, *Spirulina* could aid in recovery after strenuous physical movement. The systematic audit concludes a lack in exploration of the effect of *Spirulina* on proinflammatory cytokines like TNF-α and IL-6, as well as C-reactive protein, in people and competitors (Calella et al. 2022). C-PC reduce the creation of pro-inflammatory cytokines like interferon-gamma and TNF-α, easing inflammatory reactions. Additionally, C-PC raises levels of anti-inflammatory cytokines such as interleukin-10 in a dose-dependent way. This two-pronged method lowering provocative signals while strengthening immune defenses positions C- PC as a promising choice for boosting immune wellness and soothing irritation, particularly during strenuous exercise or sickness (Grover et al. 2021).

#### Immunomodulatory activities

Both inherent and acquired immunity decline temporarily up to 70% in the hours after intense exertion, making an ‘accessible window’ for opportunistic diseases. Effector lymphocytes, like NK cells, T lymphocytes, both auxiliary and cytotoxic, TCR dg-positive cells, and administrative T cells, are fundamental for the cell-intervened immune response. Studies reported that how *Spirulina* supplementation impacted the quantitative measures of immune reaction in high-execution competitors illuminating the delicate relationship between activity, stress, and immune reaction (Juszkiewicz et al. 2018). The variation in cytotoxic and Treg cells, after high-stress activity rely upon numerous elements, like, the strength and duration of the exercise, alongside singular reactions from competitors. The noteworthy increase in the Treg/(NK + Tdg + cytotoxic) proportion in the placebo group after recovery emphasizes a fundamental part of immune direction post-serious exercise. Athletes are known to have a more prominent occurrence of respiratory problems, particularly during serious preparation periods or competitions, with these issues regularly intensifying in winter because of natural components. These circumstance can bring about respiratory aggravation, which is impacted by a lack of balance in the body's anti-inflammatory reaction and elevated oxidative anxiety (Juszkiewicz et al. 2018). Even though athletes regularly follow a nutrient-dense eating regimen, including *Spirulina* supplements could offer additional backing by improving tissue resilience and diminishing oxidative harm during intense exercise (Calella et al. 2022).

#### Anti-anemic activities

Research findings show that, supplementing *Spirulina* to older adults with a history of anemia for 12 weeks consistently boosted mean corpuscular hemoglobin in both sexes, signaling enhanced oxygen capacity.The mean corpuscular volume and mean corpuscular hemoglobin concentration increased, pointing to improved red blood cell production. The upregulated indoleamine 2,3-dioxygenase activity and risen WBC count suggest immune function enhancement during supplementation (Selmi et al. 2011). The iron content in *Spirulina* sp*.* is noteworthy for potential benefits, especially for athletes and those engaging in rigorous activities. Maintaining adequate iron is vital for hemoglobin production, which plays a key role in transporting oxygen from the lungs to muscles. What makes the iron of *Spirulina* sp. particularly advantageous is that unlike compounds in other foods, phytates and oxalates don't curb absorption. This high bioavailability means the body can effectively utilize it*s* iron, possibly enhancing endurance and performance through better muscle oxygen delivery (Calella et al. 2022).

#### Antidiabetic activities

Over the past ten years, increasing numbers of people diagnosed with type 2 diabetes represent just one of the public health challenges faced by the developed countries, worldwide. This has been unambiguously established as a major causal risk factor for the development of cardiovascular diseases such as myocardial infarction, peripheral vascular disease, heart failure, stroke, eye disease, and neuropathy, mediated by microvascular and macrovascular complications due to raised blood glucose levels. The consumption of *Spirulina* has attracted many concerns owing to its ability to diminish blood glucose, control blood lipids and provide antioxidant effects (El-Sayed et al. 2018). In a study to evaluate these changes, Streptozotocin-induced diabetic rats were given *Spirulina* supplement, which resulted in a significant reduction of fasting blood sugar accompanied by an increase in plasma insulin and serum C-peptide levels concluding that *Spirulina* sp. may specifically enhance glycemic control and help control diabetes by possibly improving functionality of the pancreas and stimulating insulin secretion. Another study had shown that the consumption of 8 g of *Spirulina* as a beverage significantly lowered postprandial blood glucose concentrations at 90–120 min post-consumption in healthy adults (Lympaki et al. 2022). A clinical trial demonstrated that daily supplementation with 2 g of *Spirulina* for two months improved fasting plasma glucose and postprandial glucose levels in individuals with type 2 diabetes (Rezaiyan et al. 2023). Additionally, a systematic review concluded that *spirulina* supplementation can have beneficial effects on fasting blood glucose and blood lipid profiles in individuals with type 2 diabetes (Hatami et al. 2021). Hyperglycemia affects lipid membranes and is one of the molecular hallmarks that promote glucose output from the liver and ROS generation in muscle and adipose tissue. This blood sugar reducing action of *Spirulina* is probably related to its phenolic compounds and phycocyanin extract which has a potential effect on voltage-dependent K + channel expression as seen in mouse pancreatic β-cells (El-Sayed et al. 2018). Conversely, glibenclamide appears to exert only a small effect on β-cell function. In addition, fasting blood glucose and HOMA-IR were greatly improved (p ≤ 0.05), indicating enhanced insulin sensitivity compared with the glibenclamide or control group suggesting the potential hypoglycemic and insulin-sensitizing properties of *Spirulina* sp. or its components. The excessive number of fiber in *Spirulina* may also affect glucose absorption by delaying it, thereby decreasinng blood sugar levels. Multiple biochemical mechanisms, like the adenylate cyclase/cAMP pathway and the phosphatidylinositol pathway, as well as membrane depolarization are involved in *S. platensis* inducing insulin secretion. Proteins obtained from *Spirulina* sp. have important benefits in improving glucose metabolism and insulin resistance by facilitating glucose uptake in hepatocytes, and the liver to process glucose better. Glycogen is formed as a result in addition to the synthesis of key enzymes such as hexokinase as well as pyruvate kinase. Additionally, three peptides from *S. platensis* can inhibit the activity of enzymes like α-amylase, α-glucosidase, and dipeptidyl peptidase 4 that play an important role in blood glucose regulation. Thus, these may prove useful as therapeutic agents for the treatment of type 2 diabetes (Prete et al. 2024).

In *Spirulina*; the presence of ω-6 PUFA leads to a significant reductions in various metabolic markers. For instance, the blood glucose levels decreased by 20%; triglycerides decreased by 31%; total cholesterol fell by 22%; and MDA levels, as a marker for oxidative stress, dropped by up to 56% (Guldas et al. 2020).Additionally, the methanolic extract demonstrated strong positive results regarding its inhibitory gates concerning the key carbohydrate-based enzymes, leading to 96.46% knocking out of α-amylase and 97.42% knocking out for α-glucosidase indicating the accurate management of postprandial blood glucose levels by *S. platensis* (Gheda et al. 2021).

#### Probiotic activities

Oligosaccharides extracted from *Spirulina* sp. boosted the abundance, diversity, and composition of gut microbiota, and boosted the growth of *Bacteroides*, *Escherichia*-*Shigella*, *Eubacterium* sp., *Megamonas*, *Megasphaera*, *Blautia*, *Bifidobacterium*, and *Lactobacillus* genera (Cai et al. 2022). *Bacteroides* sp. is known for breaking down complex carbohydrates and producing short-chain fatty acids that are beneficial for gut health. *Eubacterium* sp. plays a key role in maintaining tight junctions and supports the immune system. *Lactobacillus* sp. is a well-documented probiotic that supports gut health, aids digestion, and helps modulate immune responses. *Megamonas* and *Megasphaera* are both discussed as fiber fermenters and SCFA producers. The *Blautia* genus boost gut health and metabolic processes. *Bifidobacterium* is also widely recognized as a probiotic that is vital in supporting the immune system and gut health.

Gut microbiota is shown to improve metabolic health, promoting healthy glucose response and reducing inflammation. Simply combining *S. maxima* with *Bifidobacterium* and *Lactobacillus* might pave the way for a new symbiotic approach to boosting and maintaining gut microbiota homeostasis. It is also quite likely that both probiotics will enhance the effects of *Spirulina*, and the two can kickstart major improvements in metabolic health (Wang et al. 2018a, b).

#### Anti- malarial activities

*Spirulina* sp*.*, also known as *Arthrospira platensis*, has been shown to have prominent antimalarial effects. A study reported that combining *Spirulina* with artemisinin combination therapy (ACT) had better antimalarial effects than either drug alone. However, *Spirulina* is not an effective antimalarial drug on its own (Assegaf and Astuty 2019). Furthermore, studies reported *Spirulina* to be used as a platform for malaria vaccines. Research analysis determined plant *Spirulina* capsules to have the highest antimalarial activity, with an IC50 of 2.16 μg/mL (Wulandri et al. 2018).

#### Anti-obesity activities and weight loss

*Spirulina* sp. is involved in body weight regulation by reducing fat accumulation in liver, decreasing oxidative stress, and increasing insulin sensitivity and satiety (Salazar et al. 1996). Studies showed that obese individuals who took 1–2 g/ day of *Spirulina* for three months showed body weight, BMI, and waist size reductions (DiNicolantonio et al. 2020). Additionally *Spirulina* supplementation significantly reduced body weight by an average of 1.56 kg; but with a weight reduction of 2.06 kg in obese individuals, visible through body fat percentage and waist circumference (Moradi et al. 2019). Additionally, *Spirulina* in competitive wrestlers was found to impact body fat and liver enzymes levels, with preservation of the IGF-1 and follistatin level (Bagheri et al. 2022). An aqueous extract of *Spirulina* was administered to 50-day-old diabetic rats leading to an increase in weight and albumin levels, which, in contrast to the untreated diabetics, were improved for hemoglobin and blood glucose. There was also a slight loss of body mass from 160.76 g to 160.88 g with the supplements as well as a negligible loss of waist size from 40.40 to 40.39 cm. *Spirulina* sp. also promotes hepatic lipid homeostasis by inhibiting macrophage infiltration into visceral adipose tissue. The low levels of phenylalanine in *Spirulina* can trigger the production of cholecystokinin hormone which has an appetite suppressing activity (Alfadhy et al. 2022).

#### Anti-genotoxicity activities

*Spirulina* sp., contribute to repair of DNA damages, lowering the occurrence of apoptotic cells, deduction in the number of blood samples as well as changes to erythrocytes, in rabbits who suffered from lead nitrate-induced genotoxicity and cytotoxicity (Hamed et al. 2019). *Spirulina* sp. can inhibit genotoxicity caused by urethane or reciprocated cisplatin as well. When consumed orally, it has the same effect on mice organisms as in humans (Premkumar et al. 2004). Additionally, *Spirulina* sp. has been found to lower the rate of chromosome distortion made by cadmium chloride in rats but with no effect on cervical cancer cells (Aly et al. 2018).

#### Anti-thrombic activities

The phospholipid extracts—particularly those from families of phosphatidylcholine provide significant inhibitory antiplatelet effects against inflammatory stimuli such as PAF and thrombin. These results indicate that *Spirulina* sp. has potential as a food supplement, especially for preventing thrombotic and inflammatory diseases. However, further in vivo research is necessary to confirm these in vitro findings and test *Spirulina*-derived substance safety and efficacy for related medical conditions (Koukouraki et al. 2020).

#### Radioprotective activities

The radioprotective effect on mice of a CEP extract from *S. platensis* was observed utilizing the micronucleus test for judging radio-protectivity on polychromatic erythrocytes. The evidences demonstrated that the extract markedly decreased the frequency of micronuclei caused by γ-radiation. Intrestingly, γ-radiation when applied before or after treatment with CEP worked equally implying that CEP may act as protecting medium for DNA. These results suggest that CEP has potential anticlastogenic effects and can help facilitate DNA repair processes. Moreover, in rats treated with phycocyanin from *Spirulina* sp. after irradiation, shows no evidence of reductions in either dehydrogenase activity or energy-rich phosphates and overall antioxidant defenses, thus making *Spirulina* safe (Abd El Baky and El-Baroty 2013).

### Detoxifying effect

*Spirulina* sp. has been known for its potential detoxifying effects, especially in toxic ions and heavy metals. It has active substances that might help withstand heavy metal toxification or provide anti-cancer effects. This suggests that *Spirulina* sp. may be an effective means to eliminate carcinogenic arsenic and other dangerous solutes from water, food, or the environment as well (Priyanka et al. 2023). Additionally, the extract could detoxify heavy metals in damaged kidneys making it a potential therapeutic agent (Fukino et al. 1990).

### *Spirulina* in phytoremmediation and degradable Biopolymers (PHB) production

Cyanobacteria are seen to uptake high amounts of nitrogen and phosphorus, enabling them to perform well in wastewater treatment processes, achieving over 70% removal (Bhatti et al. 2023). Studies have shown that S*pirulina* can remove cadmium less than 100 mg l^−1^ from wastewater (Rangsayatorn et al. 2002*).* Additional studies showed *Spirulina* to be effective in cleaning wastewater with DDT (1,1′-(2,2,2-trichloroethane-1,1-diyl)bis (4-chlorobenzene)) (Kurashvili et al. 2018). Furthermore, studies showed the dual functionality of *S. platensis* as a potent candidate for integrated bioremediation and bioproduct generation systems. Phyco-remediation of dyestuffs in textile wastewaters is of economic, industrial, and environmental importance. The remediation of the textile dye, Direct Green 6 (DG6), could be done by *Spirulina* *platensis,* the DG6 treatment is seen to enhance production of the biopolymer, polyhydroxybutyrate. From experimental studies it was observed that both live and dead cells of *Spirulina* sp. were capable of DG6 remediation, but live cells could be re-used with no loss of remediation efficiency. Furthermore, DG6 remediation by live cells resulted in increased algal biomass and trichome lengths, and stimulated production of valuable metabolites, including PHB, antioxidants, carbohydrates and pigments (phycobilins and astaxanthin) (Moradi et al. 2024, 2021). Furthermore, the cultivation of *Spirulina* can also provide bioplastics and other food supplements, and economic analyses have shown a favourable return on investment with a payback period of just 2.6 years (Chalermthai et al. 2023).

### *Spirulina* sp. in food industries formulating products

*Spirulina* sp. has been investigated for use in increasing health-promoting properties as well as consumer/market acceptance in other functional food products. The wheat flakes added with 2% and 6% of *spirulina*, formulated to provide energy and enhance immune response and act as a healthy snack in the form of low sugar, high fiber crackers (Batista et al. 2019). Likewise, energy bars containing *spirulina* with a 5–15% concentration provide a boost of energy (Fanari et al. 2023). *Spirulina* sp*.* has so much potential to be a healthy snack for elderly persons and for baby food. These are generally enriched with *Spirulina* at 0%, 2.5%, 5%, 7.5%, 10% and 12.5% for the sake of promoting higher health and vitality and absorptive potential of the snack (Haoujar et al. 2022). Furthermore, adding 750 mg S*pirulina* sp. per 100 g of different flours make it elderly-friendly snack (Santiago-Santos et al. 2004) containing a soft texture enriched with more nutrients for easier consumption. *Spirulina*-enriched baby foods (0%, 2.5%, 5% and 7.5%) are suitable for growth and development of infants, due to their high nutrient (Madkour et al. 2012).

*Spirulina* sp. is used in nutritional pasta products to provide improved protein content and nutrient density. Additionally, the whole-grain and gluten-free pasta with 1–15% *Spirulina*, can help with digestion and gut health, and even quick-cooking time. Heart-healthy pasta may be of the vegetable fortified variety, grown with 0–4% *Spirulina* and used with various sauces (Pagnussatt et al. 2014). *Spirulina*-fortified pasta-inspired products, such as maki sushi and jerky, produce distinct flavour and antioxidant profiles (Grahl et al. 2018). For instance, in dessert, functional ice creams blended with *Spirulina* bring together sensory indulgence and health (Malik et al. 2013). Smoothie bowls enhanced with 5% *Spirulina* assist in gut health and digestion, with that creamy mouth feel suited to desserts. Lactose-free frozen treats with 0.15% *Spirulina* are ideal for lactose intolerant consumers and immune-boosting frozen desserts (0%, 0.6% and 1.2% *Spirulina*) are appealing to consumers of all ages (Agustini et al. 2016).

Finally, the functional dairy products with *Spirulina* content from 0 to 1% (Mocanu et al. 2013), that have been developed to improve plant-based fermented dairy products (for instance yogurt), supporting gut health without compromising the characteristic sourness associated with the product. The health-conscious consumer also enjoys low-fat probiotic yogurts (0.1–1% *Spirulina*) that boost immune responses (Aghajani et al. 2019), and soft cheeses containing between 0% and 1.5% *Spirulina* are also rich in calcium and protein (Agustini et al. 2016). Acidophilus milk with 0.5 and 1% *Spirulina* along with *L*. *acidophilus* and acidophilus bifidusthermophiles fermented (ABT) milk containing 3 g L^−1^ *Spirulina* sp. along with *Bifidobacteria*, *L*. *acidophilus*, and *S. thermophiles* provide additional health benefits to humans (Varga et al. 2002).

### *Spirulina* sp. in cosmetics, biofuel production and agriculture

Microalgal bioactive compounds and lipids received growing interest as potential natural substitutes for synthetic ingredients in the cosmeceutical and skincare fields. They can be added to various products, such as eyeliners, lipsticks, eyeshadows, moisturizers, cleansers, shampoos, sunscreens, and beauty masks. Bioactive compounds available in cosmetics are carotenoids, polysaccharides, peptides and vitamins. *Spirulina* sp. bioactive compounds are highly appreciated due to their antioxidant and radical scavenging effectiveness, which mitigate aging-related signs of wrinkles (Nova et al. 2024). Peptides and carotenoids give UV protection in cream and sunscreen, and polysaccharides provide glorious hydration, contributing to the stability of moisture and oil in skin (Abreu et al. 2023).

**Fig. 3:**
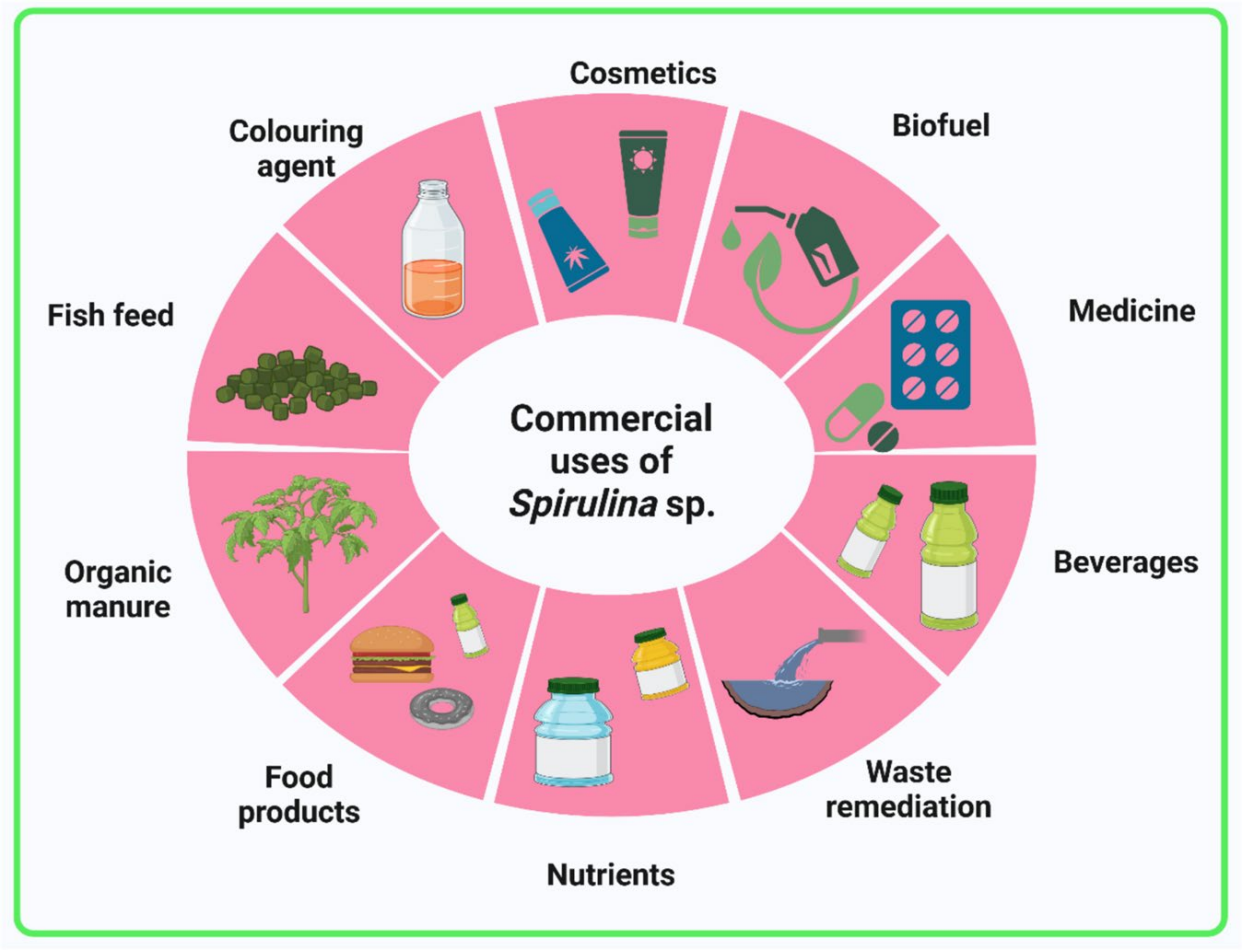
Commercial applications of *Spirulina* sp.

*Spirulina* sp. is of particular interest as a sustainable agricultural bio-stimulant, since its bioactive compounds, including phytohormones, polysaccharides, vitamins, and amino acids, can stimulate plant growth and stress tolerance. Unlike synthetic fertilizers and pesticides today, bio-stimulants based on microalgae such as *Spirulina* are a promising alternative. For instance, *S. platensis* extracts, which are high in fatty acids, enhanced early growth stage as well as wheat and rapeseed yields. Singular or combined application of these bio-stimulants, when coupled with Egyptian clover, not only boosted further growth and yield of the pea plant, but also diminished the development of fungal pathogens. The interaction treatment significantly enhanced the shoot length, leaf number, pod formation, improved soil characteristics, and the mineral composition (Abreu et al. 2023). Additionally, *Spirulina* sp. can be used to produce biodiesel and bioethanol, making it a promising alternative to fossil fuels. *Spirulina* oil can be extracted and converted into biodiesel through a process called transesterification. Using a mixture of methanol and hexane can improve the extraction of algal oil and increase the biodiesel yield. (Pradana et al. 2018). Furthermore, *Spirulina* sp*.* is commercially used as medicine as well as fish feed, baby nutrition and as coloring agents (Fig. 3).

### *Spirulina* sp. in space

*Spirulina*, has been considered for space missions, due to its potential role in CO_2_ fixation, oxygen production, sustenance creation, and recycling. Microalgae like *Spirulina* sp. can be nurtured in bioreactors, offering an area-efficient nourishment source for astronauts. Research has demonstrated that *Spirulina* sp. can thrive in bioreactors, aboard in the ISS with similar oxygen and biomass manufacture as on Earth. Examinations on Mars-like states revealed that *Spirulina*, along with other algae, can tolerate in Martian environments, with higher development rates than on Earth. Microalgae-based life support frameworks could diminish dependence on Earth, improving the plausibility of long-haul space missions by recycling CO_2_, oxygen, water and sustenance. However, difficulties, for example, space radiation, low gravity, and other space stressors need additional investigation before these frameworks are completely actualized for missions to Mars and beyond (Sinetova et al. 2024).

### Toxicology and dosage

Although*, Spirulina* sp. is eminent for its nutritional value and generally considered safe; it might bring about several adverse reactions including allergies, headaches, insomnia, sore muscles, and sweating most in people with autoimmune illnesses or those on immunosuppressive drugs (Kumar et al. 2021). Moreover, the *Spirulina* sp. contains microcystins and β-methylamino-L-alanine (BMAA), which can pose major health risks, including liver damage, gastrointestinal disturbances, and neurodegenerative dangers, that result at Alzheimer's and Parkinson's diseases on extended uses (Trotta et al. 2022; Gogna et al. 2023). Furtermore, high dosage could lead to biochemical dysfunction and histopathological changes in major organs (El-Tantawi and Abo-Zeid 2020). Additionally, *Spirulina* may accumulate lead, mercury, or arsenic if grown in polluted water. Therefore, it should be contraindicated to anyone with phenylketonuria (PKU), as it contains phenylalanine. Furtermore, it can aggravate autoimmune diseases such as lupus or multiple sclerosis. Pregnant and breastfeeding women, as well as those on blood-thinners such as warfarin, should consult healthcare professionals before using *spirulina*, since it contains vitamin K, which may interfere with such medications. High-quality, certified products free of contaminants such as microcystins need to be selected, and dosages strictly followed in order to minimize risks from *Spirulina* (Gogna et al. 2023).

The optimal daily doses of *spirulina* range between 3 and 10 gm for adults (Gogna et al. 2023). Additionally, dosages of 2 g a day have been found effective in improving growth and reversing anemia in specific populations, such as preschool children in low-income settings. It is also likely that lower doses will be equally effective (Barennes et al. 2022).

## Conclusion

*Spirulina* sp. has significant potentialities as seen through trenches in phycochemistry and pharmacology; thereby, greatly enhancing health-promoting products. *Spirulina* sp. is a promising agent of environmental protection creating food for both people and animals is an additional feature. Furthermore, having antimicrobial, immunomodulatory, nephroprotective, cardioprotective, neuroprotective, anti-aging, anti-inflammatory, and anticancer actions are among its pharmacological characteristics of *Spirulina* sp. which boost immunity, control obesity, diabetes, and cause weight reduction, lower cholesterol; eventually, prevent heart attacks, and improve health by averting many illnesses. Although, in vitro and animal studies encourage suitable clinical research in future, to understand any unknown therapeutic efficacy completely and to determine appropriate dosages for therapeutic administration. Given that *Spirulina* sp. is a beneficial nutritional supplement with proteins, carbohydrates, essential fatty acids, vitamins, minerals, carotenes, chlorophyll *a* and c-phycocyanin, it is not yet properly utilized medically, therefore commercially. It provides a restricted level of protection against viral infections, anemia and tumor growth. As naturally occurring components with some high nutritional values are gathered, a sizable amount of biomass can be grown in abio-reactor for use as a nutraceutical at a low cost. More work is required to explore its therapeutic potential and optimize its use as a bioactive material for the prevention/control of human ailments.

## Data Availability

The datasets used and analyzed during the current study are available from the corresponding author on reasonable request.

## Abbreviations

6-OHDA: 6-Hydroxydopamine
ACE: Angiotensin-converting enzyme
ALS: Amyotrophic lateral sclerosis
ARE: Antioxidant response element
ATP: Adenosine triphosphate
BGA: Bacterial growth assay
BP: Blood pressure
CA3: Cornu ammonis area 3
C-APC: Cyanophycean-allophycocyanin
Ca-SP: Calcium Spirulan
CAT: Catalase
CO2: Carbon dioxide
COX-2: Cyclooxygenase-2
C-PC: Cyanophycean-phycocyanin
C-PE: C-Phycocyanin-E
CsA: Cyclosporine A
CSB3: Cytochrome B3
DMBA: 7,12-Dimethylbenz[a]anthracene
DNA: Deoxyribonucleic acid
DOPAC: 3,4-Dihydroxyphenylacetic acid
EV-71: Enterovirus 71
GLA: Gamma-linolenic acid
GPx: Glutathione peroxidase
HBV: Hepatitis B virus
HCC: Hepatocellular carcinoma
HCMV: Human cytomegalovirus
HCT116: Human colon cancer cell line
HCV: Hepatitis C virus
HDF: Human dermal fibroblast
HDL: High-density lipoprotein
HepG2: Human hepatocellular carcinoma cell Line
HFMD: Hand, foot, and mouth disease
HIV-1: Human immunodeficiency virus type 1
HO-1: Heme oxygenase-1
HOMA-IR: Homeostasis model assessment of insulin resistance
HSV-1: Herpes simplex virus type 1
HSV-2: Herpes simplex virus type 2
IL-17: Interleukin 17
IL-6: Interleukin 6
Ile-Gln-Pro (IQP): Isoleucine-glutamine-proline
K562: Human chronic myelogenous leukemia cell line
KA: Kainic acid
LA: Linoleic acid
L-AsnA: L-Asparagine
LDL: Low-density lipoprotein
MCF-7: Michigan cancer foundation-7 (a breast cancer cell line)
MDA: Malondialdehyde
MIC: Minimum inhibitory concentration
MRSA: Methicillin-resistant *Staphylococcus aureus*
MTT: Methylthiazolyldiphenyl-tetrazolium bromide (used in cell viability assays)
MW: Molecular weight
NADPH: Nicotinamide adenine dinucleotide phosphate
NAFLD: Non-alcoholic fatty liver disease
NF-αB: Nuclear factor alpha-b
NF-κB: Nuclear factor kappa-light-chain-enhancer of activated B cells
NK: Natural killer cells
NOS: Nitric oxide synthase
Nrf2: Nuclear factor erythroid 2-related factor 2
NSP12: Non-structural protein 12 (in the context of SARS-CoV-2)
PCE: Progenitor cell enrichment
RAS: Renin angiotensin system
RNA: Ribonucleic acid
ROS: Reactive oxygen species
rRNA: Ribosomal RNA
S. maxima: Spirulina maxima
S. platensis: Spirulina platensis
SAE: Serum amyloid E
SARS-CoV-2: Severe acute respiratory syndrome Coronavirus 2
Se-PC: Selenium-phosphatidylcholine
SOD: Superoxide dismutase
sVCAM-1: Soluble vascular cell adhesion molecule-1
TAG: Triacylglycerol
TBARS: Thiobarbituric acid reactive substances
TNF-α: Tumor necrosis factor alpha
Val-Glu-Pro (VEP): Valine-glutamic acid-proline
VEGF: Vascular endothelial growth factor
WBC: White blood cell
ω-3: Omega-3
ω-6: Omega-6
